# Direct reprogramming of fibroblasts into neural stem cells by single non-neural progenitor transcription factor Ptf1a

**DOI:** 10.1038/s41467-018-05209-1

**Published:** 2018-07-20

**Authors:** Dongchang Xiao, Xiaoning Liu, Min Zhang, Min Zou, Qinqin Deng, Dayu Sun, Xuting Bian, Yulong Cai, Yanan Guo, Shuting Liu, Shengguo Li, Evelyn Shiang, Hongyu Zhong, Lin Cheng, Haiwei Xu, Kangxin Jin, Mengqing Xiang

**Affiliations:** 1 State Key Laboratory of Ophthalmology, Zhongshan Ophthalmic Center, Sun Yat-sen University, Guangzhou, 510060 China; 2 Guangdong Provincial Key Laboratory of Brain Function and Disease, Zhongshan School of Medicine, Sun Yat-sen University, Guangzhou, 510080 China; 3 Center for Advanced Biotechnology and Medicine and Department of Pediatrics, Rutgers University-Robert Wood Johnson Medical School, 679 Hoes Lane West, Piscataway, New Jersey 08854 USA; 4 Southwest Hospital/Southwest Eye Hospital, Third Military Medical University, Chongqing, 400038 China; 5 Department of Developmental Neuropsychology, School of Psychology, Third Military Medical University, Chongqing, 400038 China; 6 Present Address: Herbert Irving Comprehensive Cancer Center, Columbia University Medical Center, Room 312B, 1130 St. Nicholas Avenue, New York, New York 10032 USA

## Abstract

Induced neural stem cells (iNSCs) reprogrammed from somatic cells have great potentials in cell replacement therapies and in vitro modeling of neural diseases. Direct conversion of fibroblasts into iNSCs has been shown to depend on a couple of key neural progenitor transcription factors (TFs), raising the question of whether such direct reprogramming can be achieved by non-neural progenitor TFs. Here we report that the non-neural progenitor TF Ptf1a alone is sufficient to directly reprogram mouse and human fibroblasts into self-renewable iNSCs capable of differentiating into functional neurons, astrocytes and oligodendrocytes, and improving cognitive dysfunction of Alzheimer’s disease mouse models when transplanted. The reprogramming activity of Ptf1a depends on its Notch-independent interaction with Rbpj which leads to subsequent activation of expression of TF genes and Notch signaling required for NSC specification, self-renewal, and homeostasis. Together, our data identify a non-canonical and safer approach to establish iNSCs for research and therapeutic purposes.

## Abstract (web summary)

Fibroblasts can be reprogrammed into induced neural stem cells (iNSCs) using transcription factors expressed in neural progenitors. Here the authors show that Ptf1a, which is normally expressed in postmitotic neurons, can reprogram fibroblasts to iNSCs through Notch independent interaction with Rbpj.

## Introduction

Neurodegenerative diseases including Alzheimer’s disease (AD), Huntington’s, and glaucoma have become a global threat to human health. Traditional treatment attenuates disease progress but is overall ineffective since lost cells are not replenished in the lesion. Endogenous neurogenesis is insufficient for replenishment and results in only very limited self-repair in these diseases. Current focus of regenerative medicine emphasizes on how to generate a large number of neurons, glias or their progenitors that have the ability to integrate and function in the affected tissues, thereby providing a promising approach to lesion repair. At present, clinical application of human embryonic stem cells (ESCs) or induced pluripotent stem cells (iPSCs) has been undermined by their tumorigenic risk^1,2^. By contrast, neural stem cells (NSCs) have proven to be a safe cell resource that is not tumor prone^3,4^ and therefore provide a powerful strategy to patient-specific cell replacement therapies. They also provide a useful tool for drug discovery and in vitro disease modeling^5^.

Somatic cell reprogramming is a valuable tool for deriving patient-specific NSCs. Recent work has demonstrated that mouse and human somatic cells can be reprogrammed to transdifferentiate into induced NSCs (iNSCs)/neural progenitor cells by defined tissue-specific transcription factors (TFs)^6–9^ and/or chemicals^10,11^. In most cases of TF-induced iNSCs, reprogramming is achieved by Sox2 alone or Sox2 in combination with various other TFs^12^. More recently, a single zinc-finger TF, Zfp521, has been shown to directly reprogram human fibroblasts into iNSCs^13^. Thus, it appears that iNSC generation by TF-induced somatic cell reprogramming critically depends on Sox2 or Zfp521, which are normally expressed in proliferative neural progenitors and are key regulators of neurogenesis in vivo^14–17^. In fact, Sox2 has been postulated as a master regulator of direct iNSC reprogramming^12^. This then begs the question of whether neural progenitor TFs are the necessity for such direct reprogramming and whether it can be achieved by non-neural progenitor TFs.

Previously, we and others have identified numerous TFs, which are expressed in mitotic progenitors and/or postmitotic cells during retinal development, and have key roles in controlling retinal cell specification and differentiation^18^. We were interested in finding out whether any of these progenitor TFs and non-progenitor TFs was capable of transdifferentiating fibroblasts into iNSCs or functional neurons. Ptf1a (pancreas TF-1α) is a basic helix-loop-helix (bHLH) TF that has an indispensable role in the development of retina, cerebellum, spinal cord, and pancreas^19–23^. Here we report that unlike other typical reprogramming TFs of iNSCs, Ptf1a is selectively expressed in postmitotic precursors in the central nervous system (CNS). Moreover, unlike a number of other retinal TFs that we tested, ectopic expression of Ptf1a directly converts mouse and human fibroblasts into self-renewable and tripotent iNSCs with high efficiency. This reprogramming activity requires Notch-independent interaction between Ptf1a and Rbpj, as well as subsequent activation of expression of TF genes and Notch signaling involved in NSC homeostasis. Further, transplantation of Ptf1a-reprogrammed iNSCs improves cognitive function of AD mouse models.

## Results

### Expression of Ptf1a in non-neural progenitor cells in the CNS

In the developing CNS, Ptf1a has a limited expression pattern and has an essential role in specifying a few neuronal cell types^19,22–25^. Previously, it has been shown to be transiently expressed in postmitotic neural precursors in the retina and spinal cord^19,22^. Indeed, at E12.5, immunolabeling with an anti-Ptf1a antibody revealed very few cells co-expressing Ptf1a and the pan-proliferation marker Ki67 in the retina, spinal cord, cerebellum, and hindbrain (Supplementary Fig. 1a), indicating that Ptf1a is mostly absent from dividing neural progenitor cells in the CNS. In agreement with this, RNA sequencing (RNA-seq) data show that there is only low expression of *Ptf1a* but high expression of TF neural progenitor markers *Sox2* and *Pax6* in the E14.5 mouse retina, and that *Ptf1a* is absent from the mouse SCR029 NSCs, whereas both *Sox2* and *Pax6* are highly expressed in NSCs compared with mouse embryonic fibroblasts (MEFs) (Supplementary Fig. 1b). Similarly, *Ptf1a* transcripts exist in very low abundance in E11.5–E18 mouse CNS compared with that of TF neural progenitor markers *Pax6*, *Olig2*, and *Zfp521*, as determined by the mouse ENCODE transcriptome project (Supplementary Fig. 2). These results suggest that Ptf1a is a non-neural progenitor TF that is unlikely involved in the generation of NSCs in vivo.

### Reprogramming of MEFs by Ptf1a into self-renewable iNSCs

**Fig. 1:**
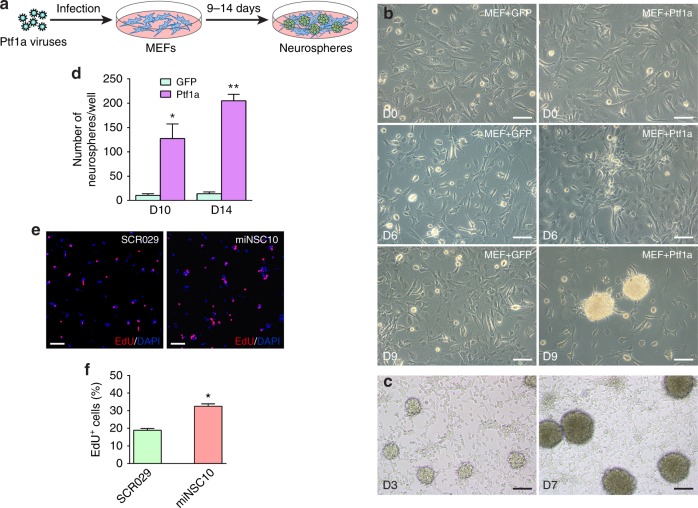
Ptf1a directly converts mouse embryonic fibroblasts (MEFs) into self-renewable neurospheres. **a** Schematic depicting the procedure to generate neurospheres from MEFs by Ptf1a lentiviruses. MEFs were prepared from E12.5 C57BL6/J mouse embryos and infected with tet-on Ptf1a lentiviruses at day 0. Neurosphere-like structures gradually appeared in 9–14 days when cultured in N3 medium containing EGF, bFGF, and doxycycline. **b** Morphological changes of MEFs infected with control GFP or Ptf1a lentiviruses. Numerous cell clusters could be seen in Ptf1a virus-infected MEFs by day 6 in culture, but rarely in GFP virus-infected MEFs. From day 9 onward, many typical neurosphere-like structures formed in Ptf1a-transduced MEFs, whereas in GFP-transduced MEFs there were only few cell aggregates, which were morphologically different from typical neurospheres. **c** Dissociated cells from collected primary neurospheres formed secondary neurospheres in 3 days in culture and morphologically homogeneous larger neurospheres by day 7. **d** Quantification of Ptf1a-induced neurospheres. MEFs (4 × 10^4^) were seeded into each well of 12-well plates, infected with Ptf1a or GFP viruses, and neurosphere-like structures in each well were then counted at day 10 and 14 following virus infection. There were significantly more neurospheres in Ptf1a-induced samples at day 10 and 14. Data are presented as mean ± SD (*n* = 3). Asterisks indicate significance in unpaired two-tailed Student’s *t*-test: *P < 0.005, **P < 0.0001. **e** EdU-labeling of Ptf1a-induced iNSCs (miNSC10) and control NSCs (SCR029). Cells were counterstained with nuclear DAPI. **f** Quantification of EdU-labeled cells. Higher percentage of proliferative cells was found in miNSC10 than in control SCR029 NSCs. Data are presented as mean ± SD (*n* = 3). The asterisk indicates significance in unpaired two-tailed Student’s *t*-test: *P < 0.0001. Scale bars, 80 μm (**b**), (**e**) and 93.1 μm (**c**)

Given the demonstrated reprogramming activities of some TFs, the expression of Ptf1a in postmitotic neurons suggests a possibility that it may be able to convert fibroblasts into mature and functional neurons. However, repeated attempts to use Ptf1a to reprogram MEFs into differentiated neurons by established procedures all failed. We then tested whether Ptf1a had the ability to directly reprogram MEFs into iNSCs, albeit somewhat counterintuitively given the expression of Ptf1a almost exclusively in postmitotic precursors. However, when MEFs were infected with doxycycline (Dox)-inducible Ptf1a lentiviruses and cultured in the N3 medium containing epidermal growth factor (EGF), basic fibroblast growth factor (bFGF), and Dox, they started to change morphology and form clusters at day 6, from which numerous neurospheres emerged by day 9 (Fig. 1a, b, d). Control MEFs infected with Dox-inducible green fluorescent protein (GFP) lentiviruses barely formed any normally shaped neurospheres (Fig. 1b, d). When multiple Ptf1a-induced neurospheres were collected as a mixture, dissociated, and replated, they rapidly formed new secondary neurospheres starting from day 3 (Fig. 1c), indicating their self-renewal ability.

We picked primary neurospheres individually to clonally derive multiple reprogrammed mouse iNSC (miNSC) cell lines (Supplementary Fig. 3). We seeded them in separate plate wells in the presence of Dox and found that cells with NSC morphology grew gradually from adhered neurospheres. They were further expanded and passaged in the presence or absence of Dox for 30 generations. At about passage 10, all miNSC lines ceased to generate neurospheres, became homogeneous, and grew in a monolayer as typical NSCs would (Supplementary Fig. 3). Consistent with the self-renewal capability of each clonally derived miNSC line, we found that ~32.6% of miNSC10 cells were pulse-labeled by EdU, which was more than that (18.9%) of the wild-type mouse NSC line SCR029 (Fig. 1e, f). Thus, miNSCs reprogrammed from MEFs by Ptf1a were proliferative and self-renewable. By contrast, MEFs infected with GFP lentiviruses failed to generate neurospheres immunoreactive for typical NSC markers and the few spheroids likely formed spontaneously looked abnormal and lacked any ability to expand in culture (Supplementary Fig. 4).

**Fig. 2:**
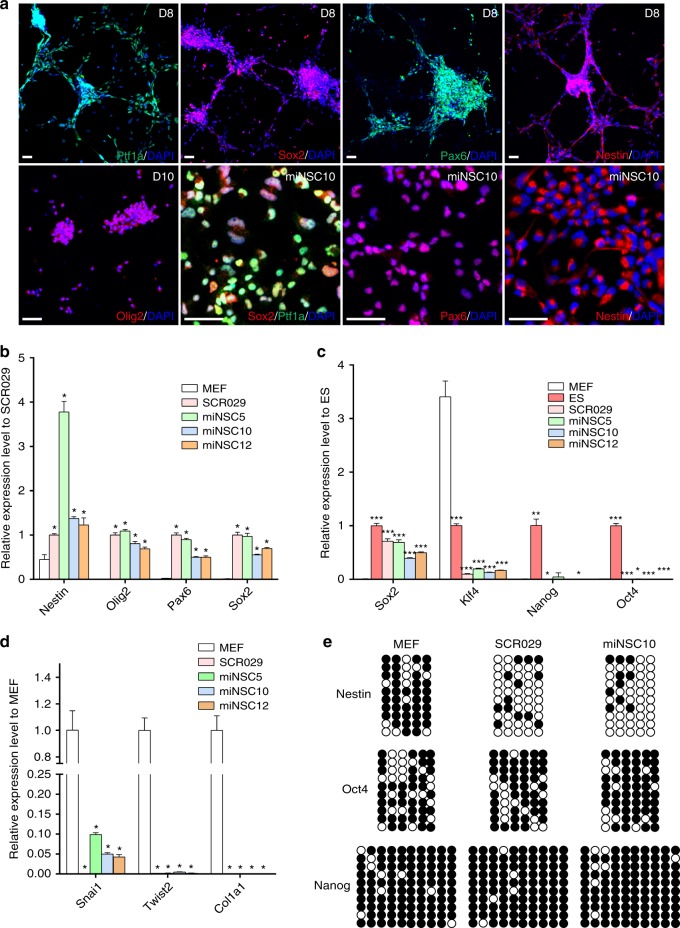
Characterization of Ptf1a-reprogrammed iNSCs. **a** Neurospheres induced from MEFs by Ptf1a at day 8 and 10 were immunoreactive for Ptf1a, Sox2, Pax6, Nestin, or Olig2. Monolayered miNSC10 cells were also immunoreactive for Ptf1a, Sox2, Pax6, or Nestin. Cells were counterstained with nuclear DAPI. Scale bars, 80 μm. **b** qRT-PCR analysis showed that in comparison with MEFs, there was a great increase in expression of *Nestin*, *Olig2, Pax6*, and *Sox2* genes in Ptf1a-derived miNSC5, miNSC10, and miNSC12 cells, as well as in control SCR029 NSCs. Data are presented as mean ± SD (*n* = 4). Asterisks indicate significance in unpaired two-tailed Student’s *t*-test: *P < 0.0005. **c** qRT-PCR assay showed that compared with mouse ESCs, there was only minimal or no expression of pluripotent factor genes *Klf4*, *Nanog*, and *Oct4* in miNSC and control NSC cells. As a pluripotent factor gene and also NSC marker gene, *Sox2* exhibited high levels of expression in both miNSC and ES cells. Data are presented as mean ± SD (*n* = 4). Asterisks indicate significance in unpaired two-tailed Student’s *t*-test: *P < 0.05, **P < 0.005, ***P < 0.0001. **d** As revealed by qRT-PCR assay, expression of fibroblast cell marker genes *Snai1*, *Twist2*, and *Col1a1* was dramatically downregulated in miNSC cells. Data are presented as mean ± SD (*n* = 4). Asterisks indicate significance in unpaired two-tailed Student’s *t*-test: *P < 0.0001. **e** DNA methylation status was examined in the promoter regions of *Nestin*, *Oct4*, and *Nanog* genes. Filled and empty circles represent methylated and unmethylated CpGs, respectively. Compared with MEFs, the promoter region of *Nestin* became hypomethylated in SCR029 and miNSC10 cells; however, the promoter regions of Oct4 and *Nanog* remain hypermethylated

To confirm that Ptf1a indeed directly induced miNSCs from MEFs, we performed immunofluorescent staining using antibodies against several NSC markers. This revealed strong expression of exogenous Ptf1a in neurospheres, which in turn induced marked expression of NSC marker proteins Sox2, Pax6, Olig2, and Nestin (Fig. 2a). In monolayered miNSCs, the expression of Sox2, Pax6, and Nestin remained high (Fig. 2a). Consistent with protein expression levels, quantitative reverse transcriptase PCR (qRT-PCR) assays demonstrated that the expression level of *Sox2*, *Pax6*, *Olig2*, and *Nestin* transcripts was greatly elevated in miNSC5, 10, and 12 lines compared with MEFs, just like in the control mouse NSC line SCR029 (Fig. 2b). By contrast, there was only minimal or no expression of pluripotent factor genes *Oct4*, *Nanog*, and *Klf4* in miNSC lines compared with mouse ESCs (Fig. 2c). Moreover, as a result of cell-fate change, the expression of MEF marker genes *Snai1*, *Twist2*, and *Col1a1* was drastically reduced in miNSC lines (Fig. 2d). In agreement with active transcription of the *Nestin* gene in miNSCs, its promoter was hypomethylated in miNSC and control SCR029 cells but hypermethylated in MEFs (Fig. 2e). However, the promoters of *Oct4* and *Nanog* were hypermethylated in all three cell types (Fig. 2e), consistent with the absence of pluripotent factor gene expression in these cells.

Six3 is a TF marker for ventral forebrain and retinal progenitor cells^26,27^. We found by semi-qRT-PCR and qRT-PCR that there was a significant increase of *Six3* expression in Ptf1a-reprogrammed miNSCs compared with MEFs (Supplementary Fig. 5a, b). To visualize Six3-positive progenitors in Ptf1a-induced neurospheres, we bred Six3-Cre transgenic driver mice with R26R-YFP reporter mice to obtain R26R-YFP; Six3-Cre embryos (Supplementary Fig. 5c). When MEFs prepared from these embryos were infected with Ptf1a lentiviruses, they formed neurospheres with many but not all cells positive for both yellow fluorescent protein (YFP) and Nestin (Supplementary Fig. 5d), suggesting a possibility that Ptf1a induced some stem cells characteristic of forebrain and retinal progenitors.

**Fig. 3:**
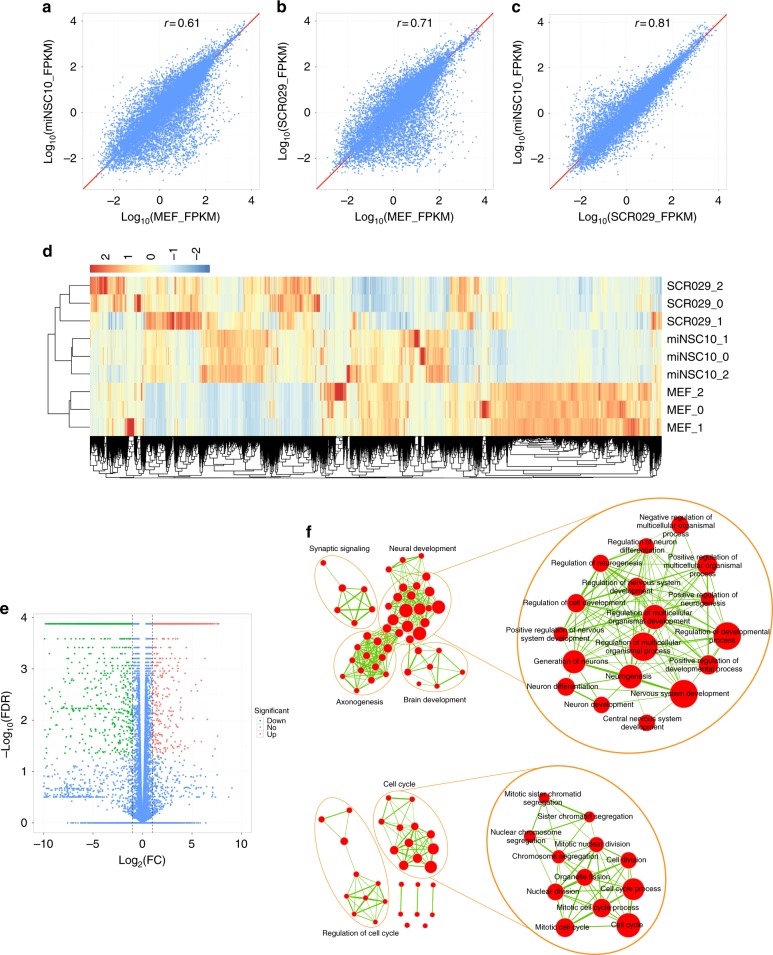
Global gene expression profiles of iNSCs, control NSCs, and MEFs. **a**–**c** Pairwise scatter plot analysis of the global gene expression profiles of miNSC10, SCR029, and MEF cells. The transcriptome of each cell type was profiled by RNA-seq analysis. Gene expression levels (FPKM) are depicted in log_10_ scale. Pearson’s correlation coefficients (*r*) are indicated. **d** Heat map of the *z*-transformed gene expression values in miNSC10, SCR029, and MEF cells. miNSC10 and SCR029 cells are classified into the same hierarchical cluster. **e** Volcano plot (significance vs. fold change) of significantly altered genes (fold change ≥ 2 and FDR < 0.05) between miNSC10 and MEF cells. **f** Gene ontology (GO) enrichment analysis of the upregulated genes between miNSC10 and MEF cells. The upregulated genes were analyzed for GO term enrichment by gene-set enrichment analysis (GSEA). The result was visualized on a network of gene sets (nodes) connected by their similarity (edges). Node size represents the gene-set size and edge thickness represents the degree of overlap between two gene sets. Depicted are the two major groups of enriched gene sets

To further confirm the NSC identity of the miNSCs, we profiled transcriptomes of miNSC10, SCR029, and MEF cells by RNA-seq analysis. Scatter plots of gene expression levels among miNSC10, SCR029, and MEF cells revealed that miNSC10 and SCR209 cells were similar to each other but were highly divergent from MEFs (Fig. 3a-c). In agreement with these results, hierarchical cluster analysis also showed a high degree of similarity between miNSC10 and SCR029 cells but great difference between them and MEFs (Fig. 3d). Numerous genes were downregulated or upregulated in expression levels in miNSCs compared with MEFs (Fig. 3e; Supplementary Data 1). We performed gene-set enrichment analysis (GSEA) of the upregulated genes followed by network visualization (Fig. 3f). Two major groups of clustered networks emerged. One was enriched for neural development-relevant GO (Gene Ontology) terms such as neurogenesis, neuron differentiation, nervous system development, regulation of neurogenesis, brain development, and axonogenesis. The other was enriched for cell cycle-relevant GO terms including cell division, mitotic cell cycle, nuclear division, sister chromatid segregation, and regulation of cell cycle. These data are consistent with miNSCs as NSCs and their capacity to proliferate and self-renew. In agreement with this, further analyses showed that miNSC10, SCR029, NS5^28^ (mouse ES cell-derived NSCs), and ciNSC^10^ (NSCs chemically induced from MEFs) cells are, albeit somewhat distinct, more similar to each other than to MEFs (Supplementary Fig. 6).

Ptf1a is expressed in dividing pancreas primordia and required for the generation of multipotent pancreatic progenitor cells^20,21^. Moreover, pancreas stem cells and NSCs are both characterized by Nestin expression^29^. Thus, Ptf1a-reprogrammed miNSCs might also represent pancreas stem cells. This possibility was tested by semi-qRT-PCR assay for a series of markers for pancreatic progenitors and differentiated cells. None of the tested progenitor markers (*Pdx1*, *Neurog3*, *Nkx2-2*, *Neurod1*, *Mafa*, *Isl1*, and *Foxa2*) showed any expression in miNSCs, and miNSCs were also negative for expression of the insulin genes (*Ins1* and *Ins2*) and glucagon gene (*Gcg*) (Supplementary Fig. 7), effectively ruling out Ptf1a-reprogrammed miNSCs as pancreas stem cells.

### Tripotency of Ptf1a-reprogrammed miNSCs

**Fig. 4:**
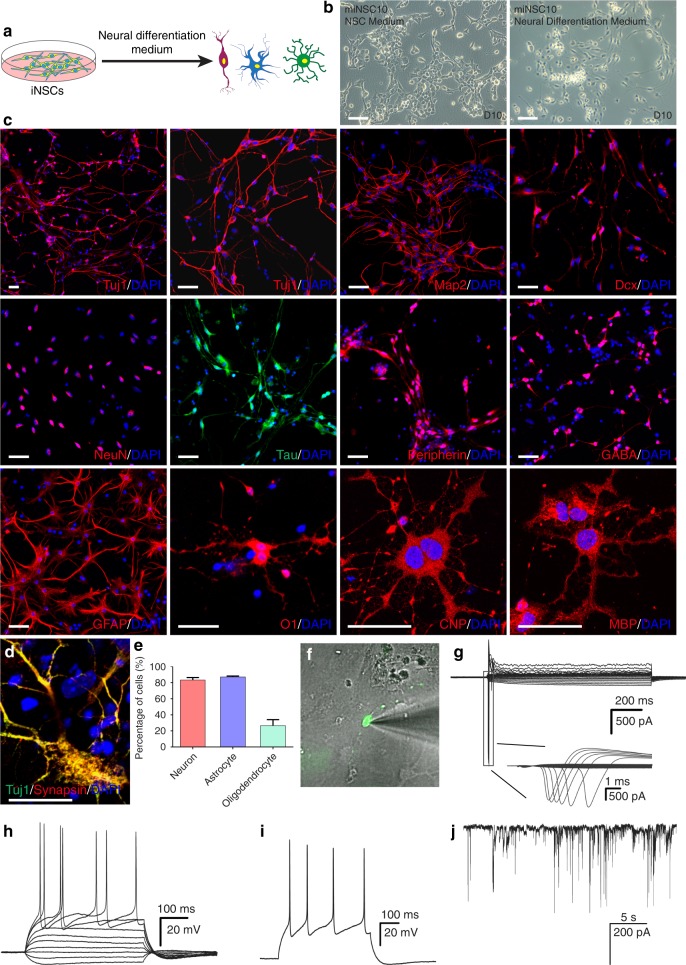
In vitro differentiation potential of Ptf1a-reprogrammed miNSCs. **a** Schematic showing that miNSCs reprogrammed directly from MEFs by Ptf1a are able to differentiate into neurons, astrocytes, and oligodendrocytes under proper culture conditions. **b** miNSC10 cells underwent drastic morphological changes to form neuron-like cells when the culture medium was switched from NSC medium to neural differentiation medium. **c** miNSC10 cells could be differentiated into neurons immunoreactive for Tuj1, Map2, Dcx, NeuN, Tau, Peripherin, or GABA. They were also capable of differentiating into astrocytes (immunoreactive for GFAP) and oligodendrocytes (immunoreactive for O1, CNP, or MBP). **d** In vitro differentiated neurons were immunoreactive for both Tuj1 and synapsin. Cells in **c** and **d** were counterstained with nuclear DAPI. **e** Quantification of Map2 + neurons, GFAP+ astrocytes, and O1+ oligodendrocytes differentiated from miNSC10 cells under different differentiation conditions. **f** A merged micrograph showing a typical GFP-tagged neuron differentiated from miNSCs that was chosen for patch-clamp recording. **g** Voltage-clamp recordings indicated fast activated and inactivated inward sodium currents as well as outward potassium currents on a differentiated neuron. **h** Current-clamp recordings revealed action potential responses of the differentiated neuron under current injection. **i** Multiple action potentials were induced after depolarization of the neuron. **j** Spontaneous postsynaptic currents recorded from an in vitro differentiated neuron. Scale bars, 80 μm (**b**) and 40 μm (**c**, **d**)

We asked whether miNSCs reprogrammed by the non-neural progenitor TF Ptf1a had the potential to differentiate into neurons, astrocytes, and oligodendrocytes as typical NSCs would (Fig. 4a). When cultured in neural differentiation medium, in a few days, miNSCs underwent robust morphological changes with decreased cell body size and pervasive neurite extension (Fig. 4b). At 2–3 weeks of culture, many cells differentiated into neurons immunoreactive for Tuj1, Map2, Dcx (doublecortin), NeuN, Tau, peripherin, or GABA (Fig. 4c). Quantification at 3 weeks showed that 83.3% of all cells were positive for Tuj1 (Fig. 4e). Furthermore, different miNSC lines (miNSC5, 10 and 12) displayed a similar capacity to differentiate into neurons immunoreactive for Tuj1 or Map2 (Supplementary Fig. 8). Under astrocyte differentiation condition, 87.2% of differentiated cells developed into glial fibrillary acidic protein (GFAP)-immunoreactive astrocytes (Fig. 4c, e). In oligodendrocyte differentiation medium, miNSCs were able to differentiate into oligodendrocytes immunoreactive for O1, CNP, or MBP, and 26.6% of differentiated cells were O1-positive (Fig. 4c, e). Thus, Ptf1a-reprogrammed miNSCs are tripotent, being able to differentiate into neurons, astrocytes, and oligodendrocytes.

The neurons differentiated from miNSCs exhibited active membrane properties. Following 2 weeks of differentiation, most of the neurons (seven out of nine) generated potassium currents and small sodium currents but no action potentials, suggesting that they were functionally immature. At 3 weeks, whole-cell patch-clamp recording showed that some differentiated neurons (4 out of 11) had typical sodium and potassium currents, and exhibited multiple action potential responses (Fig. 4f–i). Moreover, some neurons (6 out of 26) showed spontaneous postsynaptic currents (Fig. 4j). Consistent with this observation, double-immunostaining revealed that numerous neurons were labeled by punctate synapsin staining (Fig. 4d), indicating the formation of synaptic connections between differentiated neurons. Therefore, Ptf1a-reprogrammed miNSCs are able to differentiate into functional neurons.

**Fig. 5:**
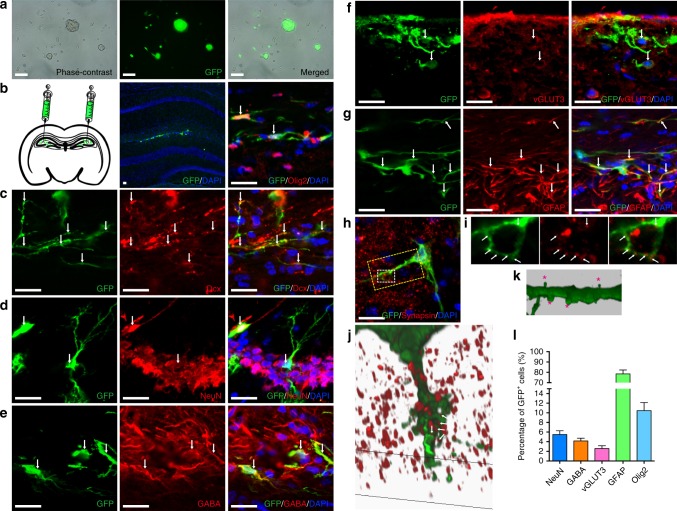
In vivo differentiation potential of Ptf1a-reprogrammed miNSCs. **a** miNSC10 cells were infected with lentiviruses containing CMV promoter-driven GFP and neomycin. After screening with G418, most miNSC cells expressed GFP as shown. **b** GFP-tagged miNSC10 cells were injected into the hippocampus region of 2-month-old mice. One month after transplantation, GFP+ cells were found successfully integrated into the tissue. Some of them were immunoreactive for the oligodendrocyte cell marker Olig2. **c**–**f** Transplanted GFP + cells immunoreactive for neuronal cell markers Dcx, NeuN, GABA, or vGLUT3. **g** Transplanted GFP+ cells co-expressing the astrocyte cell marker GFAP. **h** Immunolabeling for the presynaptic protein marker synapsin. **i** Single confocal plane images of the region outlined by the small rectangle in **h** show direct contact between the GFP + dendrite and synapsin + presynaptic terminals of the surrounding endogenous cells. **j** The 3D surface of confocal *z*-stacks of the region outlined by the large rectangle in **h** shows the formation of synaptic connections with endogenous cells. **k** The 3D surface of *z*-stacks of a dendrite shows the development of dendritic spines with typical head–neck structures (indicated by asterisks). Cells in **b**–**h** were counterstained with nuclear DAPI. Large arrows in **b**–**g** point to representative colocalized cells and/or processes. Small arrows in **i** and **j** indicate direct contact between neurites and multiple synapsin + presynaptic teminals. **l** Quantification of GFP + cells that are immunoreactive for NeuN, GABA, vGLUT3, GFAP, or Olig2. Scale bars, 80 μm (**a**) and 20 μm (**b**–**h**)

We assessed the potential of Ptf1a-reprogrammed miNSCs to differentiate into the three cell lineages in vivo. miNSCs were tagged by GFP-expressing lentiviruses and transplanted into the hippocampal region of adult mice by injection (Fig. 5a, b). One to 1.5 months after transplantation, immunolabeling showed that GFP-tagged miNSCs survived, integrated, and differentiated in the mouse hippocampus (Fig. 5b). Some of them differentiated into mature-looking neurons with multiple processes that were immunoreactive for Dcx, NeuN, GABA, or vGLUT3 (Fig. 5c–f). Some others differentiated into GFAP-positive astrocytes or Olig2-positive oligodendrocytes (Fig. 5b, g). Quantification of colocalized cells revealed that there were 5.5%, 4.7%, 2.6%, 78.4%, and 10.5% of GFP-positive cells that differentiated into NeuN+ , GABA+ , vGLUT3+ , GFAP+ , and Olig2+ cells, respectively (Fig. 5l). Thus, Ptf1a-reprogrammed miNSCs have the multipotency to differentiate into the three neuronal and glial cell types in vivo. In addition, we observed that the differentiated neurons developed mature dendritic spines with typical head–neck structures, and that their dendrites were in direct contact with multiple synapsin+ presynaptic terminals of the surrounding host cells (Fig. 5h–k), suggesting that miNSC-derived neurons are able to form synaptic connections and functionally integrate into the existing neuronal circuitry.

### Therapeutic effect of miNSCs on AD models

**Fig. 6:**
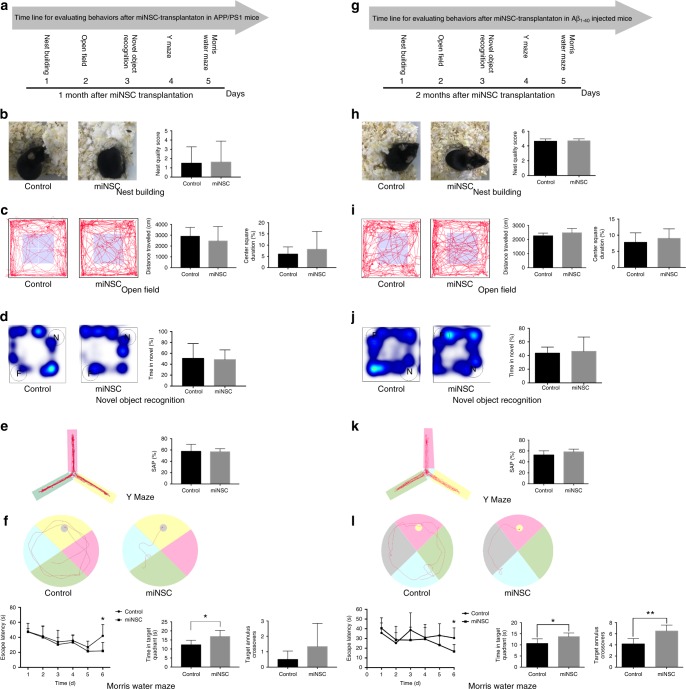
miNSC transplantation in the hippocampus improved the cognitive dysfunction of APP/PS1 and Aβ_1-40_-injured mice. **a** Time table of evaluating the behaviors of APP/PS1 mice after miNSC transplantation. The order for behavioral tests is nest-building, open-field test, novel object recognition, Y-maze test, and Morris water maze test. **b** Nest-building test. The nest-building behavior of control and miNSC-transplanted mice was assayed by assessing the nest quality after 24 h exposure to a sheet of tissue cotton. Comparing the nest quality, no statistically significant difference was observed between groups. **c** Open-field test. An illustrative example of travel pathways for a saline-injected mouse and a miNSC-transplanted mouse in the open-field test. Distance traveled and center square duration in the open-field test show no difference between the groups. **d** Novel object recognition test. An illustrative example of travel hotspot maps for a saline-injected mouse and a miNSC-transplanted mouse in the novel object recognition test. miNSC-transplanted mice show no difference in the preference for the novel. **e** Y-maze test. An illustrative example of travel pathways for a saline-injected mouse and a miNSC-transplanted mouse in the Y-maze test. Spontaneous alternation (SAP) in the Y-maze was unimproved for the miNSC-transplanted mice. **f** Morris water maze test. An illustrative example of travel pathways for a saline-injected mouse and a miNSC-transplanted mouse in the Morris water maze test. Learning curves for Morris water maze acquisition trials were obtained across a period of 6 days. Time spent in target quadrant in the Morris water maze shows that miNSC-transplanted mice spent more time in the target quadrant. Target annulus crossovers reveal that miNSC-transplanted mice show a preference for the target platform location without reaching statistical significance. **g**–**l** As in **a**–**f**, but tested after miNSC transplantation in Aβ_1-40_-injured mice. Data are presented as mean ± SD (*n* = 6–11). Asterisks indicate significance in ANOVA test: *p < 0.05, **p < 0.01

Given the vitality and tripotent differentiation potential of Ptf1a-induced miNSCs in vivo, we tested whether transplanted miNSCs had any therapeutic effect in treating murine neurodegenerative disease models. GFP-tagged miNSCs were transplanted into the hippocampus of the APP/PS1 mouse models of AD^30,31^. One month after transplantation, a series of behaviors were tested to evaluate the cognitive function of the transplanted animals (Fig. 6a). No difference in nest quality was observed between saline- and miNSC-injected mice in the nest-building test (Fig. 6b). In the next open-field test, no difference in the total distance traveled or the center time was observed between saline- and miNSC-injected mice either (Fig. 6c).

To investigate the learning and memory behavior, novel object recognition test, Y-maze test, and Morris water maze test were carried out. The results showed that between saline- and miNSC-injected mice, there was no difference in the time spent on the novel object in the novel object recognition test and no difference in the total percentage of correct spontaneous alternations (SAP) in the Y-maze test (Fig. 6d, e). However, the Morris water maze test (Fig. 6f) showed that the average escape latency was significantly decreased for miNSC-transplanted animals compared with the control group at D6 (testing day) (Fig. 6f), and that miNSC-transplanted mice spent more time in the target quadrant (Fig. 6f). Target annulus crossovers revealed that miNSC-transplanted mice appeared to have a preference for the target platform location but this preference did not reach statistical significance (Fig. 6f). Together, these results suggest that transplanted miNSCs can significantly improve spatial learning and memory of the APP/PS1 AD mouse model.

We also evaluated the therapeutic potential of miNSCs in the Aβ_1-40_ (amyloid β peptide 1–40)-injured AD mouse model, which was created by injection of Aβ_1-40_ into the hippocampus^32^. Two weeks after Aβ_1-40_ injection, we performed a series of behavioral tests to assess the cognitive function of the Aβ_1-40_-injured mice (Supplementary Fig. 9). We found that in the novel object recognition test, the time spent on the novel object was significantly decreased for Aβ_1-40_-injected mice (Supplementary Fig. 9d). In Morris water maze test (Supplementary Fig. 9f), the average escape latency was significantly increased for Aβ_1-40_-injected mice compared with saline-treated controls at D6 (Supplementary Fig. 9f) and the injected mice spent markedly reduced time in target quadrant and failed to show a preference for the target platform location (Supplementary Fig. 9f). Therefore, Aβ_1-40_-injured mice display learning and memory impairment similar to that of AD.

To test whether transplanted miNSCs produce any therapeutic effect in the Aβ_1-40_-injured AD mouse model, GFP-tagged miNSCs were transplanted into the same location 2 weeks after Aβ_1-40_ injection. Two months later, we performed the same set of behavioral tests described above (Fig. 6g). Notably, Morris water maze test (Fig. 6l) showed that the average escape latency was significantly decreased for miNSC-transplanted animals (Fig. 6l), and that miNSC-transplanted mice spent more time in the target quadrant and had a preference for the target platform location (Fig. 6l). There was no significant difference between groups in nest quality, total distance traveled and center time in open-field test, preference for novel object, and SAP in Y maze (Fig. 6h–k). Thus, transplanted miNSCs can significantly improve spatial learning and memory of both APP/PS1 and Aβ_1-40_-injured AD mouse models, producing a similar therapeutic effect as ESC-derived NSCs^33,34^.

### Dependency of Ptf1a reprogramming on Rbpj interaction

**Fig. 7:**
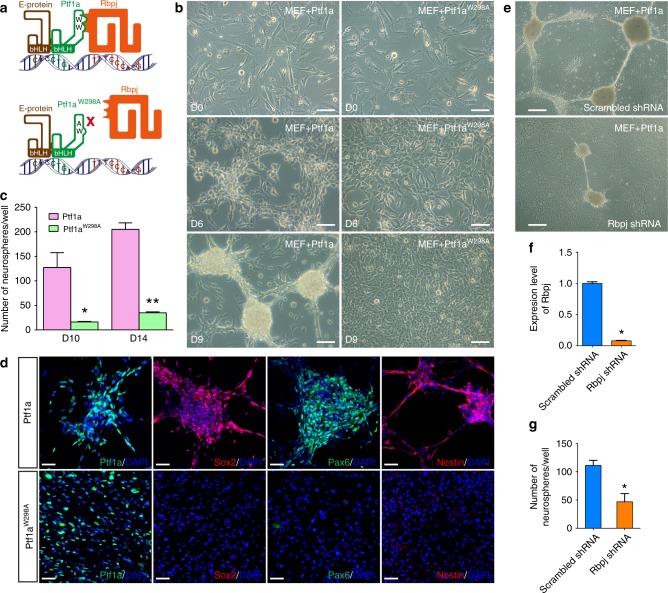
Ptf1a reprogramming activity depends on Notch-independent interaction with Rbpj. **a** Schematic depicting the trimeric DNA-binding complex formed among Ptf1a, Rbpj, and an E-protein, as well as the Ptf1a^W298A^ C-terminal mutant that lacks the ability to interact with Rbpj. **b** Unlike Ptf1a lentivirus-infected MEFs, MEFs transduced with Ptf1a^W298A^ lentiviruses failed to undergo morphological changes by day 6 or form neurospheres by day 9. **c** Quantification of neurospheres induced by Ptf1a and Ptf1a^W298A^. MEFs (4 × 10^4^) were seeded into each well of 12-well plates, infected with Ptf1a or Ptf1a^W298A^ viruses, and neurospheres in each well were then counted at day 10 and 14 following virus infection. There was a dramatic decrease of neurospheres in Ptf1a^W298A^-induced samples at day 10 and 14. Data are presented as mean ± SD (*n* = 3). Asterisks indicate significance in unpaired two-tailed Student’s *t*-test: *P < 0.005, **P < 0.0001. **d** MEFs infected with Ptf1a lentiviruses generated neurospheres at day 8 that were immunoreactive for Sox2, Pax6, and Nestin, whereas MEFs infected with Ptf1a^W298A^ viruses were negative for these NSC markers. Cells were counterstained with nuclear DAPI. **e** Neurosphere formation by Ptf1a in MEFs infected with lentiviruses expressing Rbpj shRNA or scrambled Rbpj shRNA. **f** Relative expression levels of *Rbpj* in MEFs infected with lentiviruses expressing Rbpj shRNA or scrambled Rbpj shRNA as determined by qRT-PCR assay. Data are presented as mean ± SD (*n* = 6). Asterisk indicates significance in unpaired two-tailed Student’s *t*-test: *P < 0.0001. **g** Quantification of neurospheres induced by Ptf1a in the presence of Rbpj shRNA or scrambled Rbpj shRNA. MEFs (4 × 10^4^) were seeded into each well of 12-well plates, infected with Ptf1a lentiviruses and viruses expressing Rbpj shRNA or scrambled Rbpj shRNA. Neurospheres in each well were counted at day 10 following virus infection. Data are presented as mean ± SD (*n* = 6). Asterisk indicates significance in unpaired two-tailed Student’s *t*-test: *P < 0.0005. Scale bars, 160 μm (**e**), 80 μm (**b**), and 40 μm (**d**)

It has been shown previously that Ptf1a forms a trimeric DNA-binding complex with Rbpj and an E-protein independent of Notch signaling to specify pancreatic lineages and GABAergic neurons^35,36^. We investigated whether Notch-independent interaction between Ptf1a and Rbpj is also required for the Ptf1a reprogramming activity. In MEFs, western blotting and co-immunoprecipitation assays showed that Rbpj protein was endogenously expressed, and that exogenously expressed Ptf1a could co-immunoprecipitate Rbpj (Supplementary Fig. 10). A mutant form of Ptf1a, Ptf1a^W298A^, which contains a substitution of a conserved amino acid residue tryptophan by alanine at the C-terminus, has previously been shown to disrupt Ptf1a interaction with Rbpj but not E-protein^36^ (Fig. 7a). We found that MEFs transduced with Dox-inducible Ptf1a^W298A^ lentiviruses did not undergo typical morphological changes to form cell clusters by day 6 and produced only few normal-shaped neurosphere-like aggregates by day 9–14, unlike those infected with wild-type Ptf1a viruses, which generated hundreds of neurospheres by day 9–14 in a well of 12-well plate (Fig. 7b, c). Consistent with this observation, MEFs infected with Ptf1a^W298A^ lentiviruses failed to express NSC protein markers Sox2, Pax6, or Nestin (Fig. 7d). Thus, interaction with Rbpj is a prerequisite to Ptf1a reprogramming activity. Consistent with this, knockdown of Rbpj expression in MEFs reduced the number of Ptf1a-induced neurospheres by more than twofold (Fig. 7e–g).

### Regulation of iNSC homeostasis by TFs and Notch signaling

**Fig. 8:**
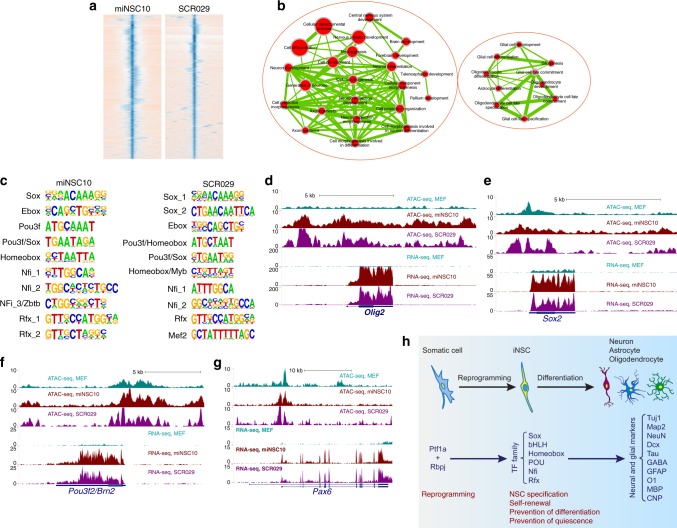
Genome-wide analysis of chromatin accessibility and gene expression in iNSCs and NSCs. **a** Heatmaps of ATAC-seq signals for miNSC10 and SCR029 cells within an 8 kb window centered around the peak summit. **b** Gene ontology (GO) enrichment analysis of the genes associated with miNSC10 ATAC-seq peaks. They were analyzed for GO term enrichment by BiNGO and the result was visualized on a network of gene sets (nodes) connected by their similarity (edges). Node size represents the gene-set size and edge thickness represents the degree of overlap between two gene sets. Depicted are two prominent groups of enriched gene sets. **c** The 10 top-ranked TF-binding motifs for both miNSC10 and SCR029 cells identified by de novo motif search in a 300-bp window centered at the peak summit. **d**–**g** Genome browser view of ATAC-seq and RNA-seq signals at the *Olig2*, *Sox2*, *Pou3f2*, and *Pax6* loci in MEF, miNSC10, and SCR029 cells. The y axis represents the number of normalized reads. **h** Schematic showing the process by which Ptf1a directly reprograms somatic cells into tripotent iNSCs and the associated molecular changes. Ptf1a must form DNA-binding complex with Rbpj to directly or indirectly activate expression of several families (Sox, bHLH, homeobox, POU, Nfi, and Rfx) of transcription factor genes involved in NSC self-renewal and maintenance

ATAC-seq profiles genome-wide accessible chromatin regions that can be bound by TFs^37^. We performed ATAC-seq analysis of miNSC10 iNSCs and SCR029 NSCs, and found that peak-associated genes are enriched for those involved in neurogenesis, neuron development, brain development, gliogenesis, and glial cell development (Fig. 8a, b), consistent with NSC features. De novo motif discovery in a 300 bp window centered at the peak summit revealed that both miNSC10 and SCR029 cells are enriched for similar sets of TF-binding motifs belonging to several families of TFs including Sox, bHLH (Ebox), Pou3f, homeobox, Nfi, and Rfx (Fig. 8c). Correspondingly, RNA-seq data show that multiple members of each of these TF gene families are upregulated or highly expressed in miNSC10 and SCR029 cells compared with MEFs (Supplementary Fig. 11a, c–f). Apart from *Sox2*, qRT-PCR validated that there is upregulation of *Sox5*, *6*, *8*, and *21* expression in miNSC10 and SCR029 cells (Supplementary Fig. 11b). In general, the chromatin accessibility profile of miNSC10 cells closely resembles that of SCR029 cells but is quite distinct from that of MEFs; and for upregulated TF genes, e.g., *Olig2* (bHLH), *Sox2*, *Pou3f2/Brn2*, and *Pax6* (homeobox), there are more peaks and/or differential peaks within these loci in miNSC10 and SCR029 cells compared with MEFs (Fig. 8d–g; Supplementary Fig. 12), indicating a more open chromatin configuration for elevated gene expression.

The six families of TFs corresponding to the most enriched motifs all contribute to maintaining NSC homeostasis such as NSC state specification, self-renewal, and prevention of quiescence and neuronal differentiation (Fig. 8h). Sox2 is required for the specification and maintenance of NSCs. It does so by binding to distal enhancers in cooperation with class three POU domain TFs, especially Pou3f2 which is also involved in specifying the neural lineage from ESCs^38,39^. Olig2 promotes NSC self-renewal by activating pro-proliferation genes, as well as inhibits premature neuronal differentiation and stem-cell quiescence by repressing genes involved in these processes^40^. Pax6 is a well-known homeobox TF essential for the determination, self-renewal, multipotency, and neurogenesis of NSCs^41,42^. The Lhx2 homeobox TF has a role in the specification of NSCs by activating *Pax6* expression, while attenuating bone morphogenetic protein (BMP) and Wnt signaling^43^. The Nfi and Rfx family members have been shown to co-occupy genomic sites with Sox2 and Pou3f2, and are essential for CNS development and quiescent state^38,44,45^.

Notch signaling is a classic cell contact-dependent signaling pathway required for NSC proliferation and self-renewal, as well as for prevention of untimely neuronal differentiation of NSCs^46^. Our RNA-seq data reveal a dramatic upregulation of Notch signaling component genes *Notch1*, *Dll1*, *Hes1*, and *Hey1* in both miNSC10 and SCR029 cells compared with MEFs and these loci also display differential ATAC-seq profiles (Supplementary Fig. 13a–d), indicating that Dll1-Notch signaling is activated in both miNSCs and NSCs for their renewal and maintenance. Consistent with the upregulation of *Notch1*, *Dll1*, and *Hes1* genes, their promoters are hypomethylated in miNSC10 and SCR029 cells compared with MEFs (Supplementary Fig. 13e). qRT-PCR assay also showed that Ptf1a induced *Notch1*, *Dll1*, and *Hes1* expression in MEFs in a time-dependent manner but had no effect on *Rbpj* expression (Supplementary Fig. 14). To determine whether Notch signaling is involved in self-renewal and maintenance of neurospheres reprogrammed by Ptf1a from MEFs, we applied the Notch signaling inhibitor *N*-[*N*-(3, 5-difluorophenacetyl)-l-alanyl]-*S*-phenylglycine *t*-butyl ester (DAPT) in the reprogramming process. We found that DAPT reduced not only the number of neurospheres but also the expression of Notch signaling effector genes *Hes1* and *Hes5* in a dose-dependent manner (Supplementary Fig. 13f–j), suggesting that Ptf1a activates Notch signaling, which in turn is required for self-renewal and maintenance of Ptf1a-reprogrammed miNSCs.

### Reprogramming of human fibroblasts by Ptf1a into iNSCs

**Fig. 9:**
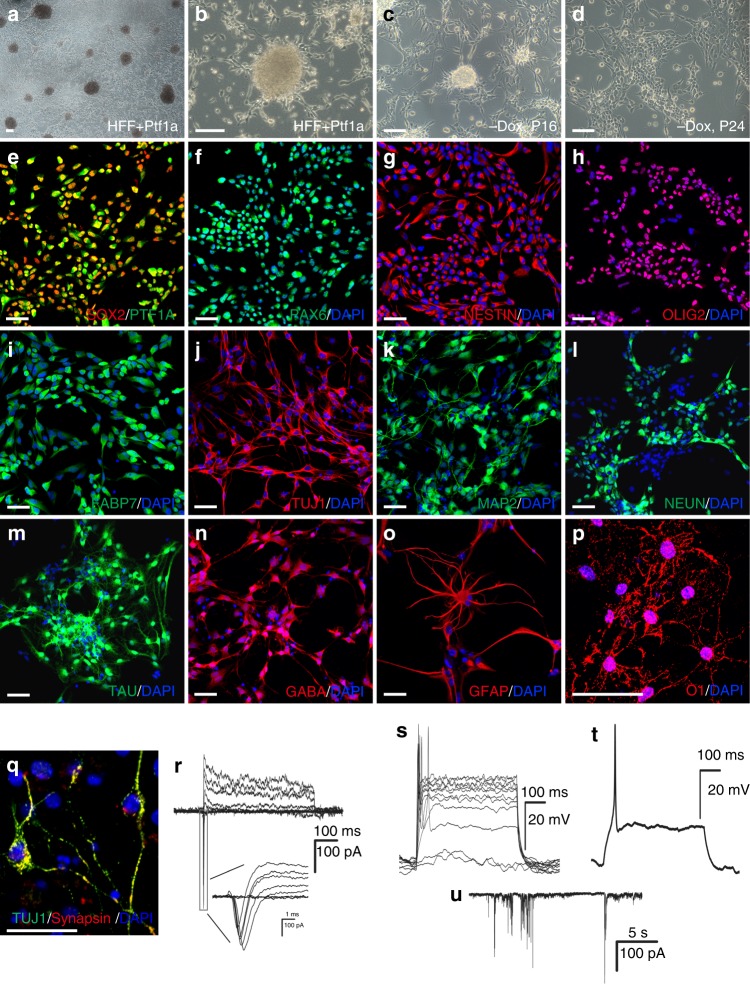
Ptf1a reprograms human foreskin fibroblasts (HFF) into tripotent iNSCs. **a**, **b** Ectopic expression of Ptf1a in HFFs by lentiviruses induced the formation of neurospheres. **c**, **d** In the absence of doxycycline (Dox), Ptf1a-induced neurosphere cells were capable of forming neurospheres before passage 20 (**c**), but lost the capacity after passage 20 and became monolayered (**d**). **e**–**i** Ptf1a-induced human neural stem cells (hiNSCs) were highly immunoreactive for PTF1A, SOX2, PAX6, NESTIN, OLIG2, and FABP7. **j**–**p** Ptf1a-induced hiNSCs were capable of differentiating into neurons immunoreactive for TUJ1, MAP2, NEUN, TAU, or GABA, astrocytes labeled by GFAP, or oligodendrocytes marked by O1. **q** Neurons differentiated from hiNSCs were immunoreactive for both Tuj1 and synapsin. Cells in **f**–**q** were counterstained with nuclear DAPI. **r** Voltage-clamp recordings indicated fast activated and inactivated inward sodium currents as well as outward potassium currents on a differentiated neuron. **s** Current-clamp recordings revealed action potential responses of a differentiated neuron under current injection. **t** An action potential was induced after depolarization of the neuron. **u** Spontaneous postsynaptic currents recorded from a differentiated neuron. Scale bars, 80 μm (**a**–**d**) and 40 μm (**e**–**q**)

Using a protocol similar to MEF reprogramming, we generated neurospheres from human foreskin fibroblasts (HFFs) by transduction of Dox-inducible Ptf1a lentiviruses (Fig. 9a, b). Interestingly, completely monolayered hiNSCs (human iNSCs) were obtained only when we expanded and passaged neurospheres in the absence of Dox (Fig. 9c, d; Supplementary Fig. 15a). At passage 20 in the absence of Dox, hiNSCs became completely monolayered without forming any neurospheres, whereas in the presence of Dox there were still many neurospheres present in the culture up to passage 32 (Supplementary Fig. 15a), suggesting that Dox-induced exogenous Ptf1a had an inhibitory effect on the generation of typical monolayered iNSCs. qRT-PCR assays revealed that the expression levels of NSC markers *SOX2*, *PAX6*, *OLIG2*, and *NESTIN* were several fold higher in hiNSCs cultured in the absence of Dox than in the presence of Dox (Supplementary Fig. 15b), providing an explanation why hiNSCs behaved more like typical NSCs in the absence of Dox. Notably, there was a higher level of endogenous *PTF1a* expression in hiNSCs cultured in the absence of Dox than in the presence of it (Supplementary Fig. 15c).

Ptf1a-reprogrammed hiNSCs barely expressed pluripotent factor genes *OCT4* and *NANOG* as determined by qRT-PCR (Supplementary Fig. 15d). By contrast, they exhibited high levels of NSC marker protein expression, including SOX2, PAX6, NESTIN, OLIG2, and FABP7(BLBP) (Fig. 9e–i). Under various differentiation conditions, hiNSCs were able to differentiate into neurons immunoreactive for TUJ1, MAP2, NEUN, TAU, or GABA (Fig. 9j–n), astrocytes immunoreactive for GFAP (Fig. 9o), or oligodendrocytes immunoreactive for O1 (Fig. 9p). Following differentiation in neuronal or glial cell differentiation media, 85.4%, 70.3%, and 33.7% of all cells developed into TUJ1-positive neurons, GFAP-positive astrocytes, and O1-positive oligodendrocytes, respectively (Supplementary Fig. 15e). Therefore, Ptf1a has a similar activity to reprogram human fibroblasts into tripotent NSCs.

We characterized the electrophysiological properties of hiNSC-derived neurons by whole-cell patch-clamp recording. Two weeks after in vitro differentiation, most of the neurons (11 out of 17) generated sodium and potassium currents, some of which looked typical of functional neurons (Fig. 9r). Some of the differentiated neurons showed action potentials (5 out of 17) and some of them (5 out of 17) exhibited spontaneous postsynaptic activities (Fig. 9s–u), suggesting that in vitro differentiated neurons can form synaptic connections. In agreement, many neurons displayed punctate immunolabeling for synapsin (Fig. 9q). Thus, similar to miNSCs, Ptf1a-induced hiNSCs are able to differentiate into mature functional neurons in vitro.

**Fig. 10:**
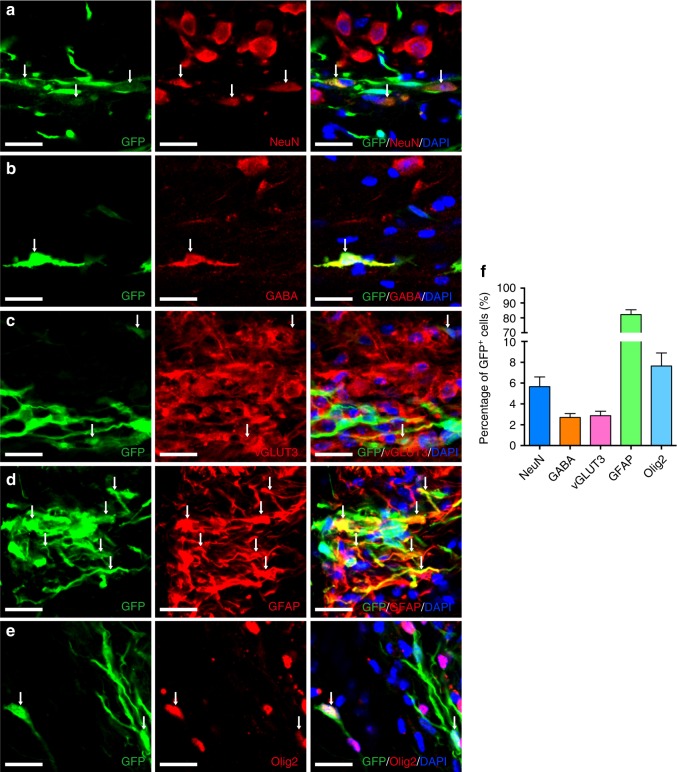
In vivo differentiation potential of Ptf1a-reprogrammed hiNSCs. **a**–**e** GFP-tagged hiNSCs transplanted into the mouse hippocampus differentiated into neurons immunoreactive for NeuN, GABA, or vGLUT3 (**a**–**c**), astrocytes immunoreactive for GFAP (**d**), or oligodendrocytes immunoreactive for Olig2 (**e**). Cells in **a**–**e** were counterstained with nuclear DAPI. Arrows point to representative colocalized cells and/or processes. **f** Quantification of GFP + cells that are immunoreactive for NeuN, GABA, vGLUT3, GFAP, or Olig2. Scale bars, 20 μm (**a**–**e**)

We further investigated the potential of Ptf1a-reprogrammed hiNSCs to differentiate into neuronal and glial cell lineages in vivo. GFP-tagged hiNSCs were transplanted into the hippocampus of adult mice and analyzed by immunostaining after 1.5 months. Similar to miNSCs, hiNSCs were able to differentiate into neurons immunoreactive for NeuN, GABA, and vGLUT3 in proportions of 5.7%, 2.7%, and 2.9%, respectively (Fig. 10a–c, f). They also differentiated into glial cells immunoreactive for GFAP and Olig2 in proportions of 82.2% and 7.6%, respectively (Fig. 10d–f). Therefore, Ptf1a-reprogrammed hiNSCs have the multipotency to differentiate into neuronal and glial lineages both in vitro and in vivo.

## Discussion

To our knowledge, this study demonstrates for the first time that a single non-neural progenitor TF has the ability to directly convert somatic cells into iNSCs, casting doubt on the hypothesis of Sox2 as a master regulator of direct iNSC reprogramming^12^. We show that Ptf1a is absent from NSCs, exhibits very low abundance in the developing CNS, and is expressed only in a small population of postmitotic cells within a few selective CNS tissues. Thus, Ptf1a can be hardly considered as a neural progenitor TF and is unlikely involved in NSC generation in vivo. Yet, unexpectedly, it displays a strong activity in directly reprogramming somatic cells into tripotent iNSCs, suggesting that neural progenitor TFs are unnecessary in driving TF-mediated reprogramming of iNSCs, and that non-neural progenitor TFs may be able to do the same as long as they have a similar driving activity. Furthermore, Ptf1a appears to be very efficient in converting fibroblasts into neurospheres. The efficiency of Sox2 alone is < 0.1%^4^. In comparison, the efficiency of Ptf1a is > 0.5% at day 14 (Fig. 1d). In previous studies of TF-mediated iNSCs, reprogramming usually involved neural progenitor TFs Sox2 or Zfp521, either alone or in combination with other TFs^12,13^. However, both Sox2 and Zfp521 present tumorigenic risks. For example, Sox2 is associated with osteosarcoma^47^, skin squamous-cell carcinoma^48^, and various other cancers^49^. Zfp521 is linked with acute B-lineage leukemia^50^ and medulloblastoma^51^. By contrast, Pft1a is inversely associated with pancreatic ductal adenocarcinoma^52^. Therefore, Ptf1a may offer a safer and more efficient solution than any other TF or combinations of TFs in generating iNSCs from somatic cells for potential therapeutic purposes.

Our work has demonstrated the feasibility of using Ptf1a-reprogrammed iNSCs as a cell replacement therapy to treat neurodegenerative diseases. First, Ptf1a has the capacity to reprogram not only murine but also human fibroblasts into self-renewable iNSCs, providing a renewable human cell source. Second, Ptf1a-induced miNSCs exhibited proper survivability and tripotent differentiation potential (neuron, astrocyte, and oligodendrocyte) when transplanted into the hippocampus of normal mice. Third, we tested whether transplanted miNSCs had any therapeutic effect in treating murine AD models and found that transplantation of Ptf1a-reprogrammed miNSCs significantly improved cognitive dysfunction, in particular spatial learning and memory of the AD mice. The therapeutic effect achieved by Ptf1a-induced miNSCs is at least compatible with that of ESC-derived NSCs^33,34^ but without the potential tumorigenic risk.

Multiple mechanisms appear to be involved in Ptf1a-mediated reprogramming of iNSCs from somatic cells. First, the reprogramming activity of Ptf1a critically depends on its interaction with Rbpj, similar to its role in determining pancreas progenitor fates. When Rbpj was knocked down by RNA interference, there were many fewer neurospheres generated. Since Rbpj is also a downstream integrator of the Notch signaling pathway, we determined whether the interaction between Ptf1a and Rbpj relies on Notch signaling. The W298A mutation of Ptf1a interrupts its interaction with Rbpj but keeps the Notch pathway intact^36^. We found that Ptf1a^W298A^ lost the ability to reprogram fibroblasts into iNSCs, demonstrating that the reprogramming activity of Ptf1a depends on its Notch-independent interaction with Rbpj.

Second, upon association with Rbpj, Ptf1a activates a few key families of downstream TF genes involved in NSC specification and homeostasis including proliferation, self-renewal, and prevention of quiescence and neuronal differentiation. RNA-seq and ATAC-seq data show that at least six families of TF genes were activated upon transdifferentiation from MEFs to iNSCs. These include Sox, bHLH, homeobox, and POU domain TF family members that have demonstrated functions in NSC generation (Fig. 8h). For instance, several Sox TFs, in particular, Sox2 were upregulated in Ptf1a-reprogrammed iNSCs. Given the ability for Sox2 alone to reprogram fibroblasts into iNSCs^4^, the reprogramming activity of Ptf1a may be mediated in part by Sox2 through direct or indirect activation by Ptf1a. All four members of the class 3 POU domain TFs were also upregulated, which presumably bind in cooperation with Sox2 to distal enhancers to regulate the specification, proliferation, and neuronal differentiation of iNSCs^14–16,38,39^. Olig2 is an upregulated bHLH TF, which has been shown to promote proliferation but prevent neuronal differentiation and quiescence of NSCs^40^. The upregulated homeobox TFs Pax6 and Lhx2 are required for the determination, proliferation, multipotency, and neurogenesis of NSCs^41–43^.

Third, Ptf1a activates Dll1-Notch signaling and perhaps other signaling pathways for efficient iNSC reprogramming. As a crucial pathway to regulate NSC proliferation and self-renewal, Notch signaling is indispensable for iNSC maintenance. Thus, neurospheres reprogrammed from MEFs by Ptf1a were decreased in the presence of Notch signaling inhibitor DAPT. Similar to its mechanism in pancreas progenitor cell expansion^53^, the Notch pathway is likely to promote iNSC proliferation in a paracrine manner. In this model (Supplementary Fig. 13k), the Ptf1a and Rbpj complex binds to *Dll1* promoter and activates its expression inside an iNSC cell; Dll1 binds to Notch receptors on a neighbor iNSC cell and activates Hes1, Hey1, and other downstream effectors. At the same time, Hes1 and Hey1 activate downstream genes to promote cell proliferation. This positive feedforward loop acts in a paracrine way to promote iNSC proliferation and expansion. Consistent with this model, our RNA-seq data reveal a dramatic upregulation of *Notch1*, *Dll1*, *Hes1*, and *Hey1* genes in iNSCs compared with MEFs. Apart from the Notch pathway, Ptf1a may activate other cell signaling pathways to promote iNSC reprogramming. For instance, many important molecules in the phosphatidyl inositol 3-kinase-Akt, Wnt, mitogen-activated protein kinase and transforming growth factor-β signaling pathways were upregulated during the process of fibroblast to iNSC conversion (Supplementary Fig. 16a), and these pathways have been proven essential in reprogramming and maintaining iPSCs and stem cells.

Lastly, there are many changes at the epigenetic and post-transcriptional levels during Ptf1a-mediated reprogramming. Aside from differential modulation of DNA methylation in the promoter regions of representative genes such as *Nestin*, *Nanog*, and *Oct4* between iNSCs and MEFs, we found that expression of many histone messenger RNAs, especially those from cluster 1, seems to be specifically regulated by Ptf1a, as these genes were downregulated in *Ptf1a*-deficient retinas^25^ but upregulated in Ptf1a-induced iNSCs (Supplementary Fig. 16b). This might reflect a general principle of how a TF modulates chromosomal structure and specifies the cell fate. Shinagawa et al.^54^ found that two of the histone variants, TH2A and TH2B (encoded by the unique genes *Hist1h2aa* and *Hist1h2ba*, respectively), could enhance the efficiency of iPSC reprogramming, indicating that functional connection with specific histones does exist. Another interesting finding is that, compared with MEFs, expression of the Dlk1-Meg3-Rian-Mirg lncRNA and microRNA cluster is downregulated more than 1600-fold in the iNSCs (Supplementary Fig. 16c). This cluster is located on chromosome 12 in mouse and accounts for approximately 10% of total microRNAs^55^. Its expression is activated in fully pluripotent mouse stem cells but repressed in partially pluripotent cells^56^; however, its role in iNSCs remains to be determined.

In summary, iNSCs have great values in patient-specific cell replacement therapies, in vitro modeling of disease pathogenesis, drug screening, and basic neurobiological studies. In this study, we have demonstrated that mouse and human fibroblasts can be successfully reprogrammed into iNSCs with a single, non-neural progenitor TF Ptf1a. The Ptf1a-derived iNSCs are clonogenic and self-renewable. They are tripotent and capable of differentiating into functional neurons, astrocytes and oligodendrocytes with high efficiency. When transplanted into the damaged hippocampus, they can significantly improve cognitive impairment of AD mouse models. The reprogramming activity of Ptf1a depends on its Notch-independent interaction with Rbpj which leads to subsequent activation of expression of TF genes and Notch and other signaling pathways involved in NSC specification, self-renewal, and maintenance.

## Methods

### Animals

Protocols and experiments in animals were approved by the Institutional Animal Careand Use Committees (IACUC) of the Zhongshan Ophthalmic Center of Sun Yat-sen University, Third Military Medical University and Rutgers University. All animals were maintained and bred in the animal facilities at these universities. Mice were fed with normal diet. The C56BL/6J mice were purchased from the Jackson Laboratory (Bar Harbor, ME) and Charles River (Beijing, China). The APP/PS1 mice were also obtained from the Jackson Laboratory.The Aβ_1-40_-injured mice were prepared as described previously^32^. Experiments with animals were performed strictly following the approved protocols.

### Cell culture

HEK293T cells (ATCC, CRL-3216) and human newborn foreskin fibroblasts (HFFs, from ATCC, SCRC-1041) were purchased from ATCC. The SCR029 cells (Chemicon, SCR029) are well-characterized mouse cortical NSCs obtained from Chemicon. These cells were tested for mycoplasma contamination before experiments and they are negative. HEK293T cells, HFFs, and MEFs were all expanded in the MEF medium [Dulbecco’s modified Eagle’s medium (DMEM, Hyclone), 10% fetal bovine serum (FBS) (Gibco), 1 × Pen/Strep (Gibco), 1 × MEM non-essential amino acids (Gibco), and 0.008% (v/v) 2-mercaptoethanol (Sigma)]. All the NSCs were plated on culture dishes pre-coated with 5 μg/ml poly-l-ornithine (Sigma) and 5 μg/ml laminin (Sigma). miNSCs and human iNSCs were cultured in the NSC medium containing the N3 medium supplemented with 10 ng/ml recombinant mouse EGF (R&D systems) and 10 ng/ml recombinant human bFGF (R&D Systems), with or without 2 ng/ml Dox (Sigma). The N3 medium contains DMEM/F12 (Life Technologies) with 1 × Pen/Strep, 25 μg/ml insulin (Sigma), 50 μg/ml Apo-transferrin (Sigma), 1.28 ng/ml progesterone (Sigma), 16 ng/ml putrescine (Sigma), and 0.52 μg/ml sodium selenite (Sigma). The SCR029 cells were cultured in the Neural Stem Cell Expansion Medium (Chemicon, SCM003) supplemented with 20 ng/ml mouse EGF, 20 ng/ml human bFGF, and 2 μg/ml heparin (Sigma).

### Viral plasmid construction and preparation of lentiviruses

The FUW-TetO vector was reported previously^57,58^. It is a lentiviral vector containing the tetracycline operator (TetO), a minimal cytomegalovirus (CMV) promoter and FUW backbone^57^. The full-length open reading frames of Ptf1a, Ptf1a^W298A^, and GFP were cloned into the EcoRI sites of FUW-TetO vector^35^. The reverse tetracycline transactivator M2rtTA^59,60^ was cloned into the EcoRI sites of the FUW backbone containing a constitutively active human ubiquitin C promoter. Lentiviruses were prepared as previously described^61^. Briefly, 293T cells were transfected with a mixture of viral plasmids and lentiviral packaging plasmids using the Lipofectamine 2000 reagent (Life Technologies) according to the manufacturer’s instruction. After 24 h of transfection, medium was replaced by 4 ml fresh medium and viral supernatants were collected at 48 and 72 h. After filtration through a 100 μm filter, viral supernatants were concentrated using an ultracentrifuge (Beckman, Optima L-100XP).

### Preparation of MEFs

E13.5–E16.5 C57BL/6J mouse embryos were collected in a 10 cm tissue culture dish containing Hanks’ balanced salt solution (HBSS; Gibco), washed briefly with HBSS, and transferred to a fresh 10 cm culture dish. The arms, legs, spinal cord, and internal organs of the embryos were removed using a pair of fine-tip forceps under a dissection microscope and any tissues above the shoulder and below hip joints were discarded. The remaining tissues were transferred to a fresh 10 cm culture plate containing 1 ml 0.25% trypsin, thoroughly minced into small pieces using a pair of surgical scissors and forceps, and incubated for 15 min at 37 °C in a CO_2_ incubator. Following the addition of 10 ml MEF medium to the plate, the digested tissues were triturated to dissociate cells by pipetting up and down several times with a 10 ml pipette. The dissociated cells were transferred into a 15 ml tube, centrifuged at 1000 r.p.m. for 5 min, and resuspended with fresh MEF media. Cells derived from three to four embryos were pooled into one 10 cm dish, cultured at 37 °C in a CO_2_ incubator, and expanded by no more than three passages.

### Generation and isolation of miNSCs and hiNSCs

To generate miNSCs, 3–4 × 10^4^ MEF cells (at passage 3) were plated on each well (12-well plate) pre-coated with poly-l-ornithine and laminin, and cultured with 1 ml MEF medium for 24 h at 37 °C in CO_2_ incubator. After removal of the MEF medium next day, 1 ml of lentiviruses and MEF medium mixture containing polybrene (10 μg/ml) was added into each well. The viruses consisted of Ptf1a, Ptf1a^W298A^, or GFP viruses, and M2rtTA viruses in a volume ratio of 1:1. To achieve optimal reprogramming efficiency, virus titration was tested for each batch of prepared viruses. Following infection of MEFs by the desired viruses for 16 h, the virus and medium mixture was removed and replaced by fresh NSC medium containing 2 ng/ml Dox. During the course of iNSC induction, the Dox-containing NSC medium was changed every 2 days. By 8–10 days after infection with the Ptf1a lentiviruses, small spheroids appeared. The size of the spheroids increased rapidly over the following 3–4 days. The procedure to induce hiNSC neurospheres from HFFs by Ptf1a is essentially the same as that for MEFs.

When neurospheres reached sufficient size, individual colonies were picked with a p20 pipette tip and replated separately into a well of 24-well plate pre-coated with poly-l-ornithine and laminin to be further expanded and characterized. After 3–5 days of expansion, the cells were dissociated with Accutase (Millipore, SF006) and transferred into a well of pre-coated 12-well plate. Subsequently, pre-coated 6-well plate, 6 cm plate, and 10 cm plate were used sequentially to expand and passage cells derived from each colony.

### RNA knockdown and Notch signaling inhibition

For *Rbpj* knockdown assay, we purchased lentiviral vectors encoding mouse *Rbpj* short hairpin RNA (shRNA) (OriGene, TL512813) or scrambled *Rbpj* shRNA (OriGene, TL512813). MEF cells were infected with a mixture of *Ptf1a* and *Rbpj* shRNA viruses for the knockdown group, and with a mixture of *Ptf1a* and scrambled *Rbpj* shRNA viruses for the control group. After 16 h, the MEF medium containing viruses was replaced with the NSC medium, which was changed every 2 days thereafter. The number of neurospheres were counted under an inverted microscope after 10 days. Total RNA was extracted from cells using the Trizol reagent (Life Technologies) and stored at − 80 ℃ for qRT-PCR assay.

The γ secretase inhibitor DAPT (from Selleck) was used to inhibit Notch signaling. After MEF cells were infected with Ptf1a viruses for 16 h, the MEF medium and virus mixture was removed and replaced with the NSC medium containing 2, 10, or 20 μm DAPT, or the same volume of dimethyl sulphoxide (Sigma) as controls. After 10 days, the number of neurospheres were counted and total RNA was extracted from cells for qRT-PCR assay.

### In vitro differentiation of miNSCs and hiNSCs

To differentiate miNSCs, 1 × 10^5^ cells were first cultured in the NSC medium for 12 h in a well of 24-well plate containing a glass coverslip coated with poly-l-ornithine and laminin. For neuronal differentiation, miNSCs were cultured in N3 medium supplemented with 2% B27 (Life Technologies), 2 mM glutamax (Gibco), and 1 × Pen/Strep for 2 days, then the medium was replaced by neurobasal-a medium (Life Technologies) supplemented with 5 μg/ml insulin, 20 ng/ml brain-derived neurotrophic factor (BDNF) (Pepro Tech), 20 ng/ml ciliary neurotrophic factor (CNTF) (Pepro Tech), 10 μM forskolin (Sigma), 25 mM l-glutamic acid, 200 mM l-glutamine, 1% B27, 1% N2 (Life Technologies), and 1 × Pen/Strep^62^. The cells were cultured for 12 more days before analysis. For generation of astrocytes, miNSCs were cultured in DMEM supplemented with 1% N2, 2 mM glutamax, and 1% FBS for 14 days^63^. Oligodendrocyte differentiation was carried out as previously described^9^. The procedure to differentiate hiNSCs into neurons, astrocytes, and oligodendrocytes was also described previously^4,63,64^.

### Immunohistochemistry and immunocytochemistry

Immunostaining of mouse embryonic tissue sections was carried out as previously described^65,66^. For immunostaining transplanted mouse brain tissues, the sections were transferred using a soft brush into wells of a 24-well plate containing 500 μl of phosphate-buffered saline (PBS) in each well, washed with 0.3% Triton-100 in PBS (PBST), blocked with 10% normal donkey serum in PBST for 1 h at room temperature, followed by staining with primary antibodies in 2% normal donkey serum at 4 °C overnight. After washing with PBST, the sections were incubated with secondary antibodies and 4′,6-diamidino-2-phenylindole (DAPI) in PBST for 1 h at room temperature, then washed with PBST, transferred onto glass slides using a soft brush, and mounted with mounting medium (Polyscience). Images were captured by a laser scanning confocal microscope (Carl Zeiss, LSM700).

For immunocytochemical staining, cells grown on glass coverslips pre-coated with poly-l-ornithine and laminin were fixed in 4% paraformaldehyde in PBS (pH 7.4) for 15 min at room temperature, washed three times with PBS, permeabilized with 0.1% Triton-100 for 10 min, blocked in 10% donkey serum for 1 h at room temperature, and then incubated at 4 °C overnight with primary antibodies diluted in 0.1% Triton-100 and 2% donkey serum. Following three rinses with PBS, the cells were incubated in secondary antibodies diluted in 2% donkey serum for 1 h at room temperature. Images were captured by a laser scanning confocal microscope (Carl Zeiss, LSM700).

The primary antibodies used in this study were as follows: chicken anti-GFP (Abcam, ab13970, 1:2000), mouse anti-Ki67 (BD PharMingen, 550609 1:20), mouse anti-vGLUT3 (Sigma, SAB5200312, 1:500), rabbit anti-synapsin1 (Abcam, ab64581, 1:500), rabbit anti-Ptf1a (Beta Cell Biology Consortium, AB2153, 1:2000), mouse anti-Sox2 (Santa Cruz, sc-365823, 1:200), rabbit anti-Pax6 (Millipore, AB2237, 1:1000), mouse anti-Pax6 (Developmental Studies Hybridoma Bank, Pax6, 1:1000), mouse anti-nestin (Millipore, MAB353, 1:1000), rabbit anti-Olig2 (Millipore, AB9610, 1:500), rabbit anti-BLBP (Millipore, ABN14, 1:500), mouse anti-Tuj1 (Sigma, T8660, 1:3000), mouse anti-Map2 (Boster Wuhan, BM1243, 1:200), chicken anti-Map2 (Millipore, AB5543, 1:3000), rabbit anti-NeuN (Millipore, ABN78, 1:500), mouse anti-NeuN (Millipore, MAB377, 1:200), mouse anti-Tau (Santa Cruz, sc-2176,1:50), rabbit anti-GABA (Sigma, A-2052, 1:3000), goat anti-Dcx (Santa Cruz, sc-8066, 1:500), rabbit anti-peripherin (Millipore, AB1530, 1:1000), rabbit anti-GFAP (DAKO, Z0334, 1:2000), mouse anti-O1 (Millipore, MAB344, 1:1000), mouse anti-MBP (Biolegend, 836502, 1:500), and mouse anti-CNP (Sigma, c5922, 1:500). The secondary antibodies used included donkey anti-rabbit, donkey anti-goat, donkey anti-mouse, and donkey anti-chicken Alexa488 IgG, Alexa594 IgG, or Alexa594 IgM (1:1000; Invitrogen). DAPI (Invitrogen) was used for nuclear counterstaining.

### Co-immunoprecipitation assay

MEFs infected with GFP or Ptf1a-Flag (flag-tagged Ptf1a) lentiviruses were collected at day 12 and lysed with the CelLytic MT Cell Lysis Reagent (Sigma, C3228) containing protease inhibitor cocktail (Roche), followed by centrifugation in a microfuge at 12,000 r.p.m. for 20 min. The protein concentration was determined by a standard bicinchoninic acid assay kit (Beyotime). Immunoprecipitation was carried out by incubating the cell lysates with anti-Flag resin (Sigma, A2220) at 4 °C overnight. After incubation, the resins were washed three times with Tris-buffered saline. The precipitates were incubated with 3 × Flag peptide elution solution with gentle shaking for 30 min at 4 °C. The eluates and whole-cell extracts (input) were separated on an 8% SDS-PAGE gel and electrotransferred to polyvinylidene fluoride membranes (Immobilon-P, Millipore). Western blotting was performed using the following primary antibodies: mouse anti-Rbpj (Santa Cruz, sc-271128, 1:500), mouse anti-Flag (Sigma, F1804, 1:5000), rabbit anti-GFP (MBL, 598, 1:2000), mouse anti-β-actin (Sigma, A5316, 1:5000), and secondary antibodies: goat anti-mouse or rabbit IgG horseradish peroxidase (KangChen, KC-MM-035, KC-RB-035, 1:5000). The membranes were incubated with enhanced chemofluorescent reagent (Pierce Biotechnology, 34095) and imaged with a digital imager (FluorChem E System, ProteinSimple).

### EdU staining

The proliferation rate of miNSC and SCR029 cells was assessed by EdU (5-ethynyl-2’-deoxyuridine, Life Technologies) labeling as described previously^67^. In brief, 1 × 10^6^ miNSC or SCR029 cells were plated on a 6 cm tissue culture dish containing a number of glass coverslips pre-coated with poly-l-ornithine, and incubated for 12 h at 37 °C in a cell culture incubator. EdU was then added into the culture medium to a final concentration of 10 μM and the cells were incubated for two more hours. The ensuing staining procedure was performed according to the manufacturer’s instructions. Images were captured with a confocal microscope.

### Bisulfite sequencing and DNA methylation analysis

Genomic DNA was isolated from MEF, miNSC10, and SCR029 cells using the DNA extraction kit (Solarbio). Unmethylated cytosines were then converted to thymines using the EpiTect Bisulfite Kit (Qiagen) according to the manufacturer’s instructions. Previously reported primers were used to amplify the promoter regions of the bisulfite-converted genes: *Nestin*, *Oct4*, *Nanog*, *Dll1*, *Notch1*, and *Hes1*^68–72^. Purified PCR products were subcloned into the PMD18T vector (Sangon Biotech) and transformed into *Escherichia coli* cells (DH5α, Takara). No fewer than 10 clones were picked for each gene and sequenced (BGI). Statistical analysis of the methylation result was performed by the BiQ Analyzer software^73^.

### Real-time qRT-PCR analysis

Total RNA was extracted from cells or tissues using the Trizol Reagent (Life Technologies) and genomic DNA contamination was removed by DNaseI (New England BioLabs). Complementary DNA was then synthesized using the AMV cDNA First Strand Synthesis Kit (New England BioLabs). qRT-PCR was performed with the LightCycler® 96 Real-Time PCR System (Roche) and all reactions were carried out in three independent biological replicates. The qRT-PCR primers used are shown in Supplementary Data 2.

### RNA-seq analysis

Total RNA was extracted from miNSC (at passage 10), SCR029, and MEF cells using the TRIzol reagent, according to the manufacturer’s instruction. Ribosomal RNA was depleted before RNA-seq library preparation. The prepared libraries were sequenced using an Illumina HiSeq 4000 sequencer (Biomarker Technologies, China). As previously described^25^, the obtained sequence reads were trimmed and mapped to the mouse reference genome (mm10) using Tophat and gene expression and changes were analyzed using Cufflinks. Hierarchical cluster and scatter plot analyses of gene expression levels were performed using the R software (http://cran.r-project.org). GSEA analysis was carried out as described^74^, which was followed by network visualization in Cytoscape using the EnrichmentMap plugin^75,76^. The accession number for the RNA-seq data reported in this study is GEO: SRP135657. In addition, we included two GEO RNA-seq datasets: GSE70872 for NS5 cells and GSE78938 for ciNSCs in our analyses.

### ATAC-seq analysis

For optimal ATAC-seq analysis, 35,000 of MEF cells, 50,000 of miNSC10 cells, and 50,000 of SCR029 cells were collected and used for the transposase reaction. ATAC-seq libraries were constructed as described previously^77^ with adaptation and index primers^37^. Library quality was assessed using the Agilent Bioanalyzer High-Sensitivity DNA kit. ATAC-seq libraries were sequenced using an Illumina HiSeq X Ten sequencer (Biomarker Technologies). The HOMER (Hypergeometric Optimization of Motif EnRichment, http://homer.ucsd.edu/homer/index.html) software suite was used to perform peak calling, determine peak position and distribution on the genome, discover de novo binding motifs, and identify peak-associated genes^78^. Peak calling was performed using an false discovery rate cutoff of 0.001 and putative peaks were determined by requiring a fourfold enrichment over the control sample and a cumulative Poisson *P*-value < 0.0001. Putative TF-binding motifs were identified by de novo motif search in a 300 bp window centered at the peak summit. GO enrichment analysis of the genes associated with ATAC-seq peaks was performed in Cytoscape using the BiNGO plugin followed by network visualization using the EnrichmentMap plugin^75,76,79^. The accession number for the ATAC-seq data reported in this study is GEO: SRP136063.

### Electrophysiological analysis

GFP-tagged miNSCs or hiNSCs (1 × 10^5^) were plated on the glial cell feeder with NSC medium in a well of 24-well plate. To optimize the differentiation procedure, 24 h before plating miNSCs or hiNSCs, 3 × 10^4^ glial cells were plated as the feeder, which were dissociated from P0 C57BL/6J mouse brain as previous described^80^. After 24 h, the medium was replaced with neuronal differentiation medium 1 (N3 medium supplemented with 2% B27, 1% N2, 2 mM glutamax, and 1 × Pen/Strep), which was replaced with neuronal differentiation medium 2 [neurobasal-a medium supplemented with 1 × Insulin-transferrin-selenium solution (Thermo Fisher), 30 ng/ml BDNF, 30 ng/ml CNTF, 30 ng/ml Nerve growth factor (Pepro Tech), 10 μM forskolin, 25 mM l-glutamic acid, 200 mM l-glutamine, 1% B27, 1% N2, and 1 × Pen/Strep]. Two to 3 weeks after differentiation, GFP-positive cells were identified with a mercury lamp equipped on an upright microscope (BX51W1, Olympus, Japan). Then, whole-cell patch-clamp recordings were performed with an EPC-10 USB amplifier (HEKA Electronics, Lambrecht, Germany). The responses of the cells were recorded with 6–9 MΩ resistance pipettes that were filled with an internal solution consisted of the following: 105 mM K-gluconate, 5 mM KCl, 5 mM NaOH, 15 mM KOH, 0.5 mM CaCl_2_, 2 mM MgCl_2_, 5 mM EGTA, 2 mM adenosine 5’-triphosphate (disodium salt), 0.5 mM guanosine 5’-triphosphate (trisodium salt), 10 mM HEPES, and 2 mM ascorbate (pH 7.2). Coverslips with adhered cells were transferred to a recording chamber and bathed in external solution containing the following: 125 mM NaCl, 2.5 mM KCl, 1 mM MgSO_4_, 2 mM CaCl_2_, 1.25 mM NaH_2_PO_4_, 26 mM NaHCO_3_, 20 mM glucose, bubbled with 95% O_2_ and 5% CO_2_. The chamber was mounted on an upright microscope equipped with a × 40 water-immersion objective and differential interference contrast (DIC) optics. The cells and recording pipettes were viewed on a monitor that coupled to a charge-coupled device camera (Evolve, Photometrics, Tucson, USA) mounted on the microscope. Oxygenated external solution was continuously perfused into the recording chamber at a flow rate of 1.5–2 ml/min by a peristaltic pump (Lead-2, LongerPump, Hebei, China). Capacitive transients were compensated via the Patch Master software (PatchMaster, HEKA) and the series resistance was compensated by ~50%. For current-clamp recording, We set the initial resting membrane potential (Vrest) to − 70 mV using a small, constant holding current and applied current pulses with a step size of 10 pA to test the ability to generate action potentials. Voltage-clamp recordings were performed directly following current-clamp recordings on the same cells. A simple step protocol from -90 mV to + 30 mV for 200 ms was applied to assess the voltage-gated sodium channels and voltage-gated potassium channels.

### Transplantation of miNSCs and immunohistochemistry

miNSCs and hiNSCs infected with the pLenti-CMV-GFP-Neo (Addgene, 17447) lentiviruses were cultured on a 10 cm tissue culture dish in the NSC medium in the presence of G418 (500 μg/ml). After 48 h in culture, GFP-tagged miNSCs survived and produced small neurospheres. These neurospheres were digested with Accutase and centrifuged, and the dissociated cells were resuspended and diluted to a final concentration of 1 × 10^5^ cells/μl. Two microliters of the cell suspension were injected into the hippocampal region (anterior-posterior, 2 mm; left–right lateral, 1.5 mm; and dorso-ventral, 2 mm) of an anesthetized C57BL/6 J mouse (8 weeks old, male or female) using a stereotaxic apparatus (RWD Life Science). The injected mouse was placed on an electric blanket until it fully awoke.

One to 1.5 months after transplantation, the animals were anesthetized and perfused by cardiac puncture with PBS followed by 4% paraformaldehyde. The brains were collected, fixed with 4% paraformaldehyde for 4 h at 4 °C with slow shaking and dehydrated in 30% sucrose for 48 h. They were then encased in tissue freezing medium (Leica) and stored at − 80 °C, followed by sectioning into 20 μm coronal sections using a cryostat (CM1950, Leica) for immunohistochemistry.

### Behavioral tests

The APP/PS1 mice (B6.Cg-Tg[APPswe,PSEN1dE9]85Dbo/Mmjax; Jackson Laboratory, Bar Harbor, ME) utilized in this study were male and at the age of 11–12 months. Two-month-old male C57BL/6J mice were used for Aβ1-40-injury as described previously^32^. The APP/PS1 and Aβ_1-40_-injured animals were transplanted with GFP-tagged miNSCs into the hippocampus as described^33^. Before the behavioral tests began, mice were placed in the experimental room for at least 30 min for acclimatization. All mice were kept in their home cages and were handled by the base of their tails at all times. The less stressful tests (nest-building test, open-field test, and novel object recognition test) were conducted before the more stressful tests (Y-maze test and Morris water maze test). So behavioral tests were conducted sequentially in the following order: nest-building test, open-field test, novel object recognition test, Y-maze test, and Morris water maze test.

Nest-building test. The nest-building behavior of the mice was assayed by assessing the nest quality after 24 h exposure to a sheet of tissue cotton. Mice were placed into a new cage with thin padding and a sheet of tissue cotton (5 × 5 cm, mean weight 2.5 g) for 24 h^81,82^. Nest-building ability was assessed after 24 h according to a 5-point rating scale^81^.

Open-field test. The open-field locomotion in a novel environment was evaluated as previously described^83,84^. The open-field apparatus was constructed of grey plywood and measured 40 × 40 cm with 30 cm-high walls. Mice were placed into the center of the open field and the movements of the mouse were recorded for 10 or 30 min using a video camera secured to the top of the apparatus and analyzed using Ethovision 11.0 (Noldus). The test apparatus was cleaned with 70% ethanol between two subjects and wiped out with clean paper towels.

Novel object recognition test. Short-term spatial memory of mice was assessed using the object location test as described previously^32,85^. In the same apparatus as in the open-field test, mice were placed for 10 min into the same quadrant of the open field with two identical objects located diagonally. After 90 min, one object was replaced by a novel one and the mice were allowed to explore for another 10 min and the time spent by the animals exploring the novel and old objects was recorded. Objects and the test apparatus were cleaned with 70% ethanol between two subjects and wiped out with clean paper towels. Videos were recorded and analyzed using Ethovision 11.0 (Noldus). To analyze the cognitive performance, a location index was calculated as (*T* novel) / (*T* novel + *T* old), where *T* novel and *T* old are the time spent exploring the novel and old objects, respectively.

Y-maze test. SAP behavior (SAB) was evaluated in a symmetrical Y Maze (3 arms, 40 × 9 cm with 16 cm-high walls). Arm choices (all four paws entering one arm) were recorded during an 8 min exploration of the mice in the Y-shaped maze. Alternation was defined by recording the order of the visited arms (A, B, or C). Overlapping triplets of 3-arm visits was counted as one complete SAP. The SAB score was calculated according to the following formula: (number of SAP)/(total number of arm visits − 2)^86^. The test apparatus was cleaned with 70% ethanol and wiped out with clean paper towels between two subjects. Videos were recorded by and analyzed using Ethovision 11.0 (Noldus).

Morris water maze test. The Morris water maze, which was widely used to analyze the spatial learning and memory, was conducted in a round white pool 150 cm in diameter and 50 cm deep^87,88^. The pool was filled up to a depth of 30 cm. The pool temperature was maintained at 25 ± 0.5 °C. The escape platform was a 25 cm^2^ plexiglass square, placed in the center of one quadrant of the pool, 15 cm from the pool’s edge and submerged 1 cm beneath the water surface. The platform remained in the same position throughout the training day and was removed from the pool during the probe test.

On the first day (visible platform), mice were placed into the water facing the wall. If the mouse reached the platform before a 60 s cutoff, it was allowed to stay on the platform for 5 s then returned to the home cage. If the mouse did not find the platform in 60 s, it was gently guided onto the platform and allowed to sit on it for 20 s before returning to the home cage. This procedure was repeated for three more trials, each starting in a different quadrant. Once the animal had completed every trial, it was dried off with a clean paper towel. The four-trial training procedure was repeated for all the mice. On the next 4 days (hidden platform), the mice were trained as on the first day but with the platform submerged. After 5 consecutive days of pre-training, the animals were tested with the platform removed. During the test, mice were placed into the water from the opposite quadrant where the platform used to be, and were tested for 60 s. Videos were recorded and analyzed using Ethovision 11.0 (Noldus). The behavioral data were statistically analyzed as described below.

### Statistics

In general, each result is obtained from three or more biological samples. Statistical analysis was performed using the GraphPad Prism 6.0 and Microsoft Excel computer programs. The results are expressed as mean ± SD for experiments conducted at least in triplicates. Unpaired two-tailed Student’s *t*-test or analysis of variance (ANOVA) test and/or Mann–Whitney *U*-test were used to assess differences between two groups, and a value of *P* < 0.05 was considered statistically significant. Repeated-measures ANOVA was used to analyze the difference of escape latency between groups in the Morris water maze test. One-way ANOVA was used to assess the difference between groups in other behavioral tests.

### Data availability

The RNA-seq and ATAC-seq data that support the findings of this study have been deposited in the NCBI Gene Expression Omnibus database under accession codes SRP135657 and SRP136063, respectively.

## Electronic supplementary material

Supplementary Information

Description of Additional Supplementary Files

Supplementary Data 1

Supplementary Data 2
