# Supplementary material for: Direct reprogramming of fibroblasts into neural stem cells by single non-neural progenitor transcription factor Ptf1a
Source: Nat Commun. 2018 Jul 20;9:2865. doi: 10.1038/s41467-018-05209-1 (PMC6054649; doi:10.1038/s41467-018-05209-1)
Supplement: Supplementary file 1 — Supplementary Information [file 41467_2018_5209_MOESM1_ESM.pdf]

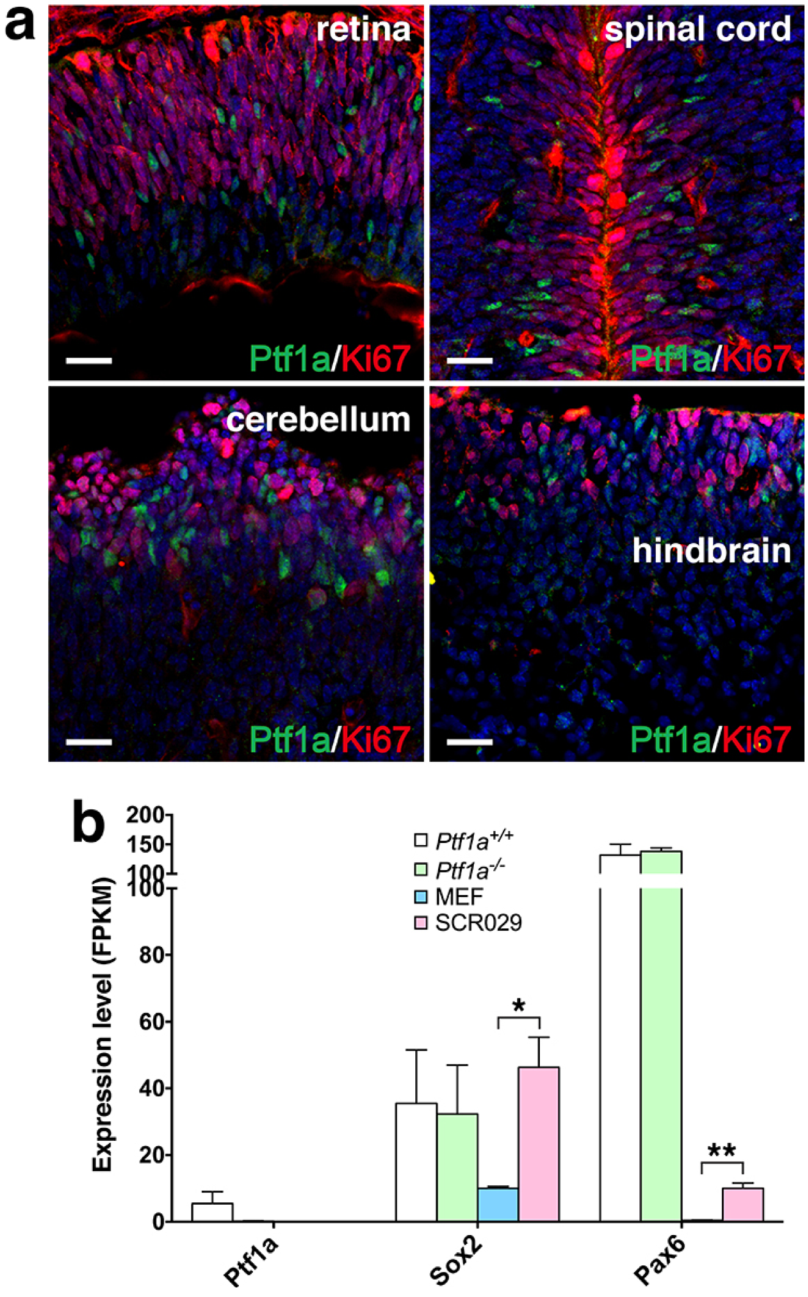

Supplementary Figure 1. Ptf1a is not expressed in mitotic neural progenitors or NSCs. (**a**) Ptf1a is expressed in a small population of postmitotic precursor cells in the developing mouse CNS. As detected by immunostaining, in E12.5 mouse embryos, Ptf1a-expressing cells (green) rarely co-express Ki67 (red), a pan-proliferative cell marker, in the retina, spinal cord, cerebellum or hindbrain. (**b**) RNA-seq data reveal that compared to neural progenitor markers *Sox2* and *Pax6*, *Ptf1a* is expressed at a low level in E14.5 mouse retinas and absent from the SCR029 NSCs. Moreover, *Ptf1a* inactivation in mice has no effect on expression levels of *Sox2* and *Pax6* in the retina. Data are presented as mean ± SD (n=3). Asterisks indicate significance in unpaired two-tailed Student's t-test: *P<0.005, **P<0.0005. Scale bars, 20 μm.

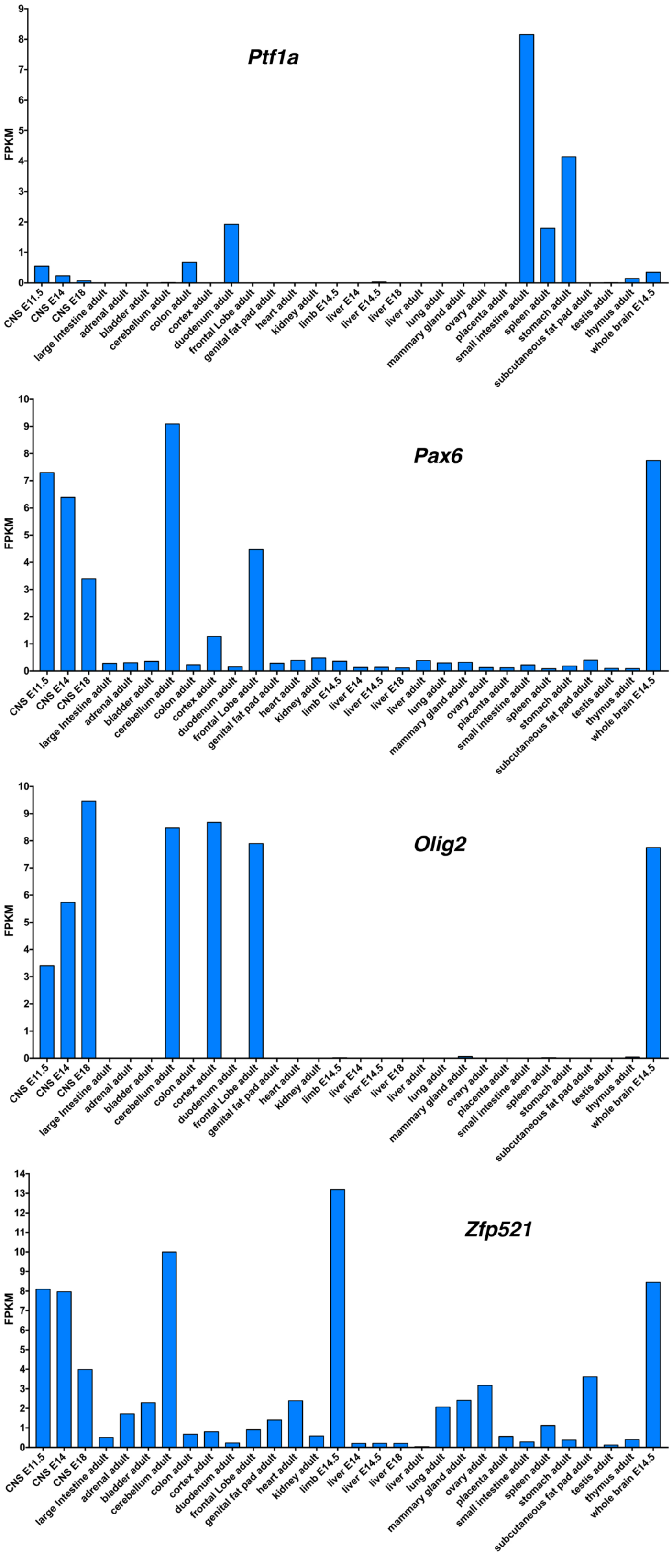

Supplementary Figure 2. Expression levels of *Ptf1a*, *Pax6*, *Olig2* and *Zfp521* in a variety of developing and adult mouse tissues as determined by the mouse ENCODE transcriptome project. Note the low abundance (FPKM) of *Ptf1a* transcript in the developing CNS compared to that of *Pax6*, *Olig2* or *Zfp521*.

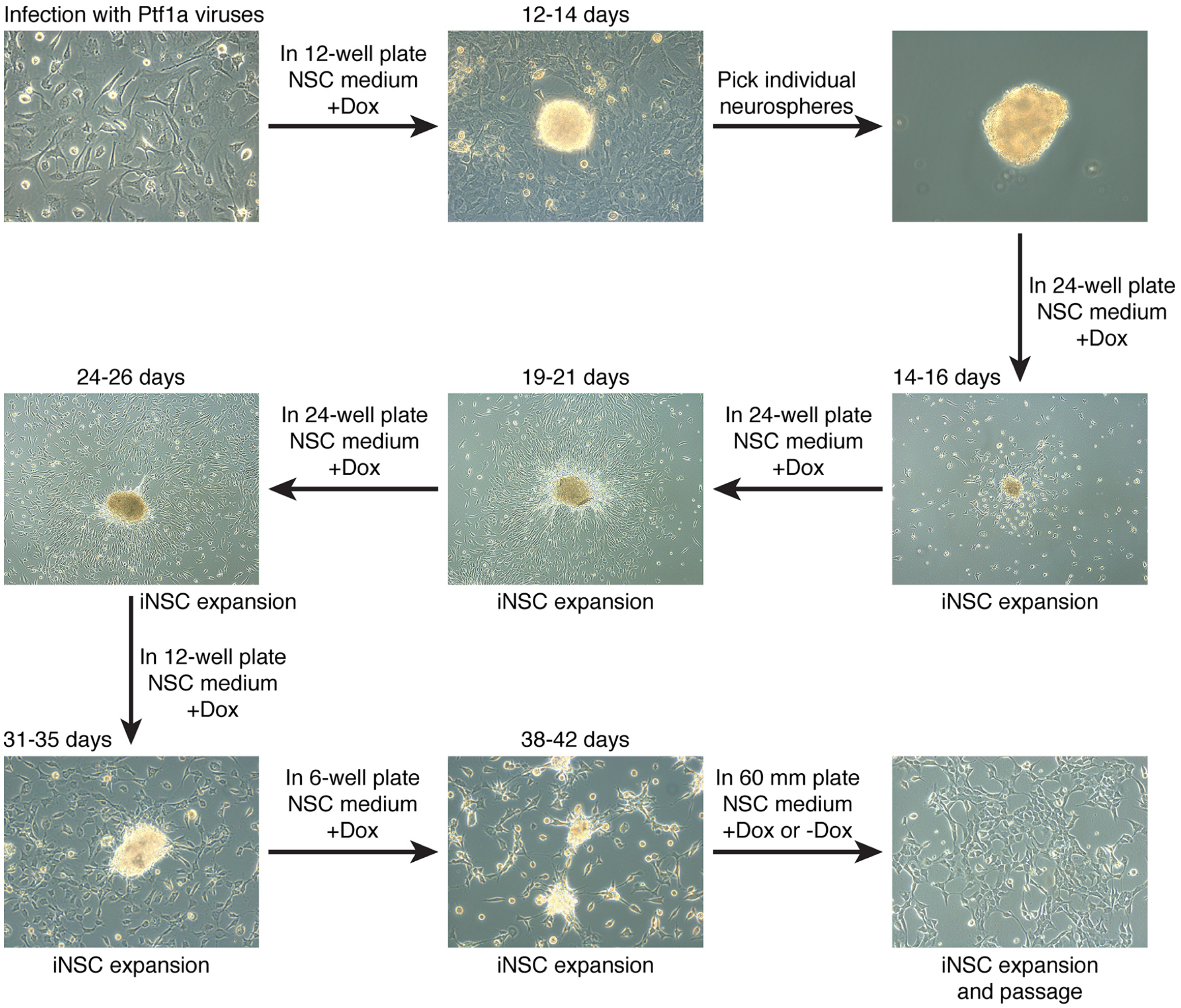

Supplementary Figure 3. Procedure to induce and expand iNSCs from MEFs by Ptf1a lentiviruses. In general, MEFs are transfected with tet-on lentiviruses encoding Ptf1a. Doxycycline (Dox) turns on ectopic expression of Ptf1a which transdifferentiates fibroblasts into neurospheres. Cells from neurospheres are gradually expanded from 24-well plates to 12-well and 6-well plates in the presence of doxycycline. These steps take about 6 weeks. Finally, the neurosphere cells are expanded and passaged in 60-mm culture dishes with or without doxycycline until they become monolayered iNSCs. The NSC medium is used in all steps.

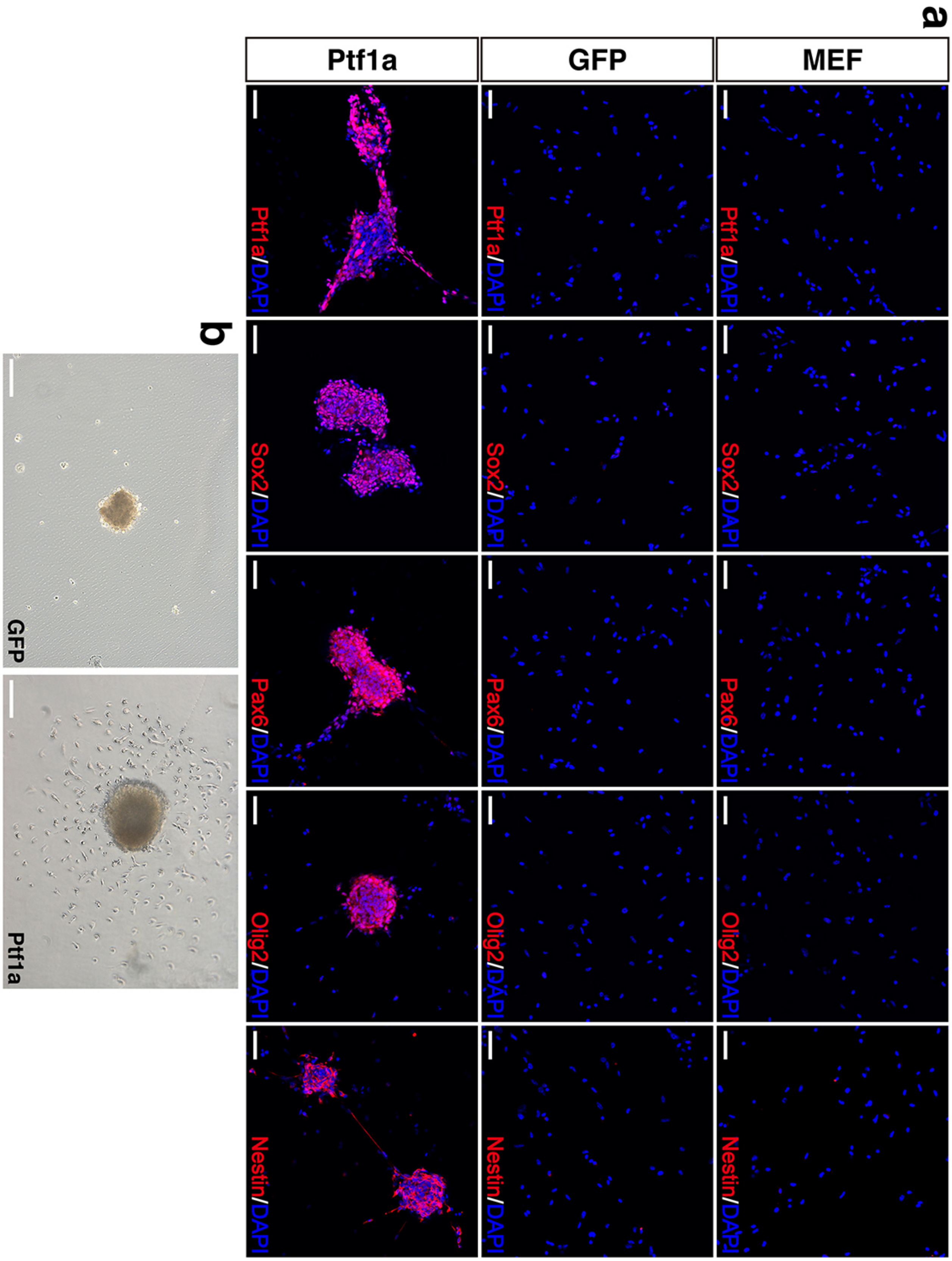

Supplementary Figure 4. Lack of neural progenitor cells in MEFs. (a) MEFs and MEFs infected with GFP lentiviruses were not immunoreactive for Ptf1a, Sox2, Pax6, Olig2, or Nestin, whereas MEFs infected with Ptf1a viruses formed neurospheres that were strongly immunoreactive for all of these protein markers. (**b**) Neurospheres reprogrammed from

MEFs by Ptf1a were able to expand in culture whereas the few spheroids formed in the presence of GFP looked abnormal and lacked the ability to expand in culture. Scale bars, 40 μm (**a**) and 80 μm (**b**).

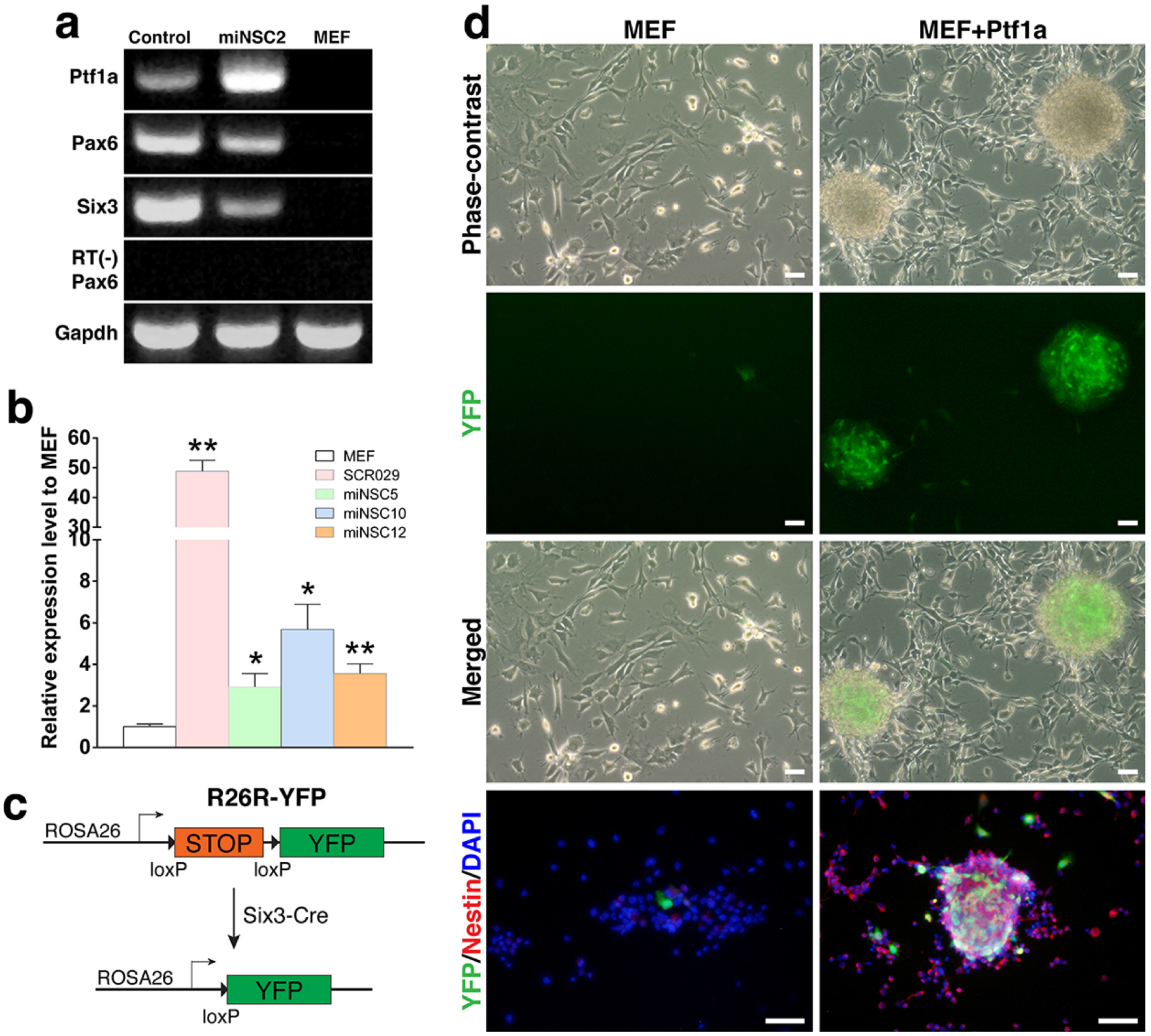

Supplementary Figure 5. Ptf1a-derived iNSCs characteristic of forebrain and retinal progenitors. (a) *Ptf1a*, *Pax6* and *Six3* are expressed in miNSC2 cells but not in MEFs. Semi-quantitative RT-PCR was performed using gene-specific primers. The control was total RNA from E15.5 mouse retinas. *Gapdh* was used as an internal control for the amount of cDNA used. RT-PCR was also performed in the absence of reverse transcriptase (RT) to serve as a negative control. **(b)** qRT-PCR analysis shows that compared to MEFs, *Six3* expression is significantly increased in miNSC5, miNSC10 and miNSC12 cells as well as in the control SCR029 NSC cells. Data are presented as mean ± SD (n=3). Asterisks indicate significance in unpaired two-tailed Student's t-test: *P<0.005, **P<0.0001. **(c)** A schema illustrating that YFP is expressed only after the stop sequence is removed from the *ROSA26* locus by *Six3* promoter-driven expression of Cre recombinase. **(d)** MEFs from the R26R-YFP; Six3-Cre mouse embryos were used to determine whether *Six3* expression was activated in the Ptf1a-reprogrammed iNSCs. High expression of YFP was seen in the neurospheres induced by Ptf1a. The NSC protein marker, Nestin, was also observed in the neurosphere. Scale bars, 80 μm.

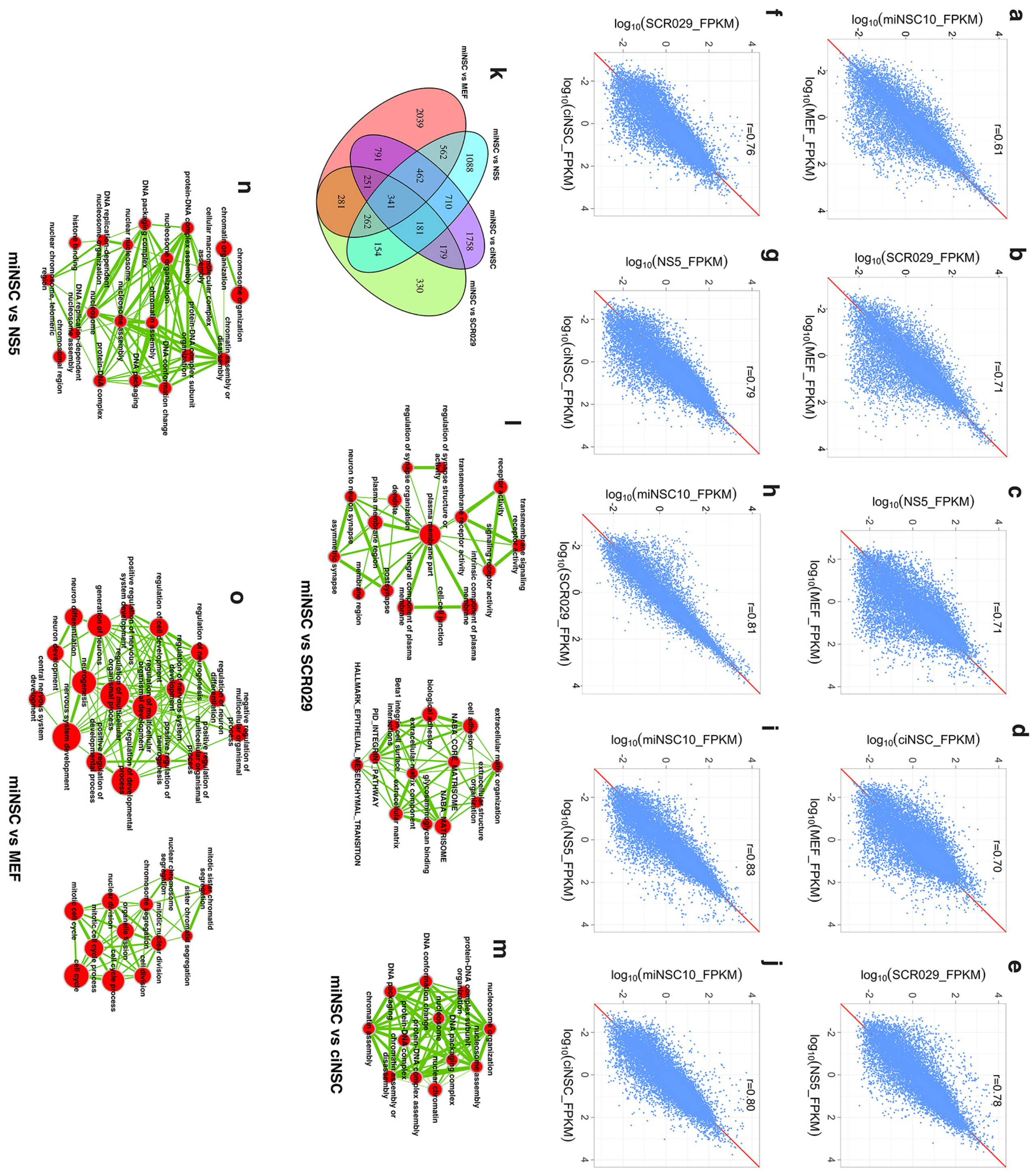

Supplementary Figure 6. Comparison of global gene expression profiles of Ptf1a-induced iNSCs, different control NSCs and MEFs. (a-j) Pairwise scatter plot analysis of the global gene expression profiles of miNSC10, SCR029, NS5 (mouse ES cell-derived NSCs), ciNSC (NSCs chemically induced from MEFs), and MEF cells. The transcriptome of each cell type was profiled by RNA-seq analysis. Gene expression levels (FPKM) are depicted in $\log_{10}$ scale. Pearson correlation coefficients (r) are indicated. (**K**) Venn diagram of differentially expressed genes shared between miNSC10 and other NSC or MEF cells. (**l-o**) Gene ontology (GO) enrichment analysis of the upregulated genes between miNSC10 and other NSC or MEF cells. The upregulated genes were analyzed for GO term enrichment

by gene set enrichment analysis (GSEA). The result was visualized on a network of gene-sets (nodes) connected by their similarity (edges). Node size represents the gene-set size and edge thickness represents the degree of overlap between two gene sets.

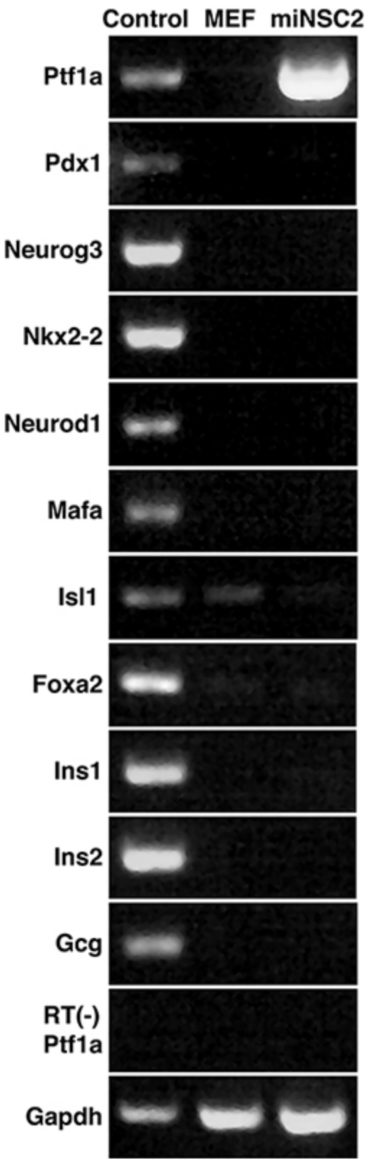

Supplementary Figure 7. Semi-quantitative RT-PCR assay reveals that pancreatic marker genes are not induced in Ptf1a-reprogrammed iNSCs. The control was total RNA from the adult mouse pancreas. *Gapdh* was used as an internal control for the amount of cDNA used. RT-PCR was also performed in the absence of reverse transcriptase (RT) to serve as a negative control.

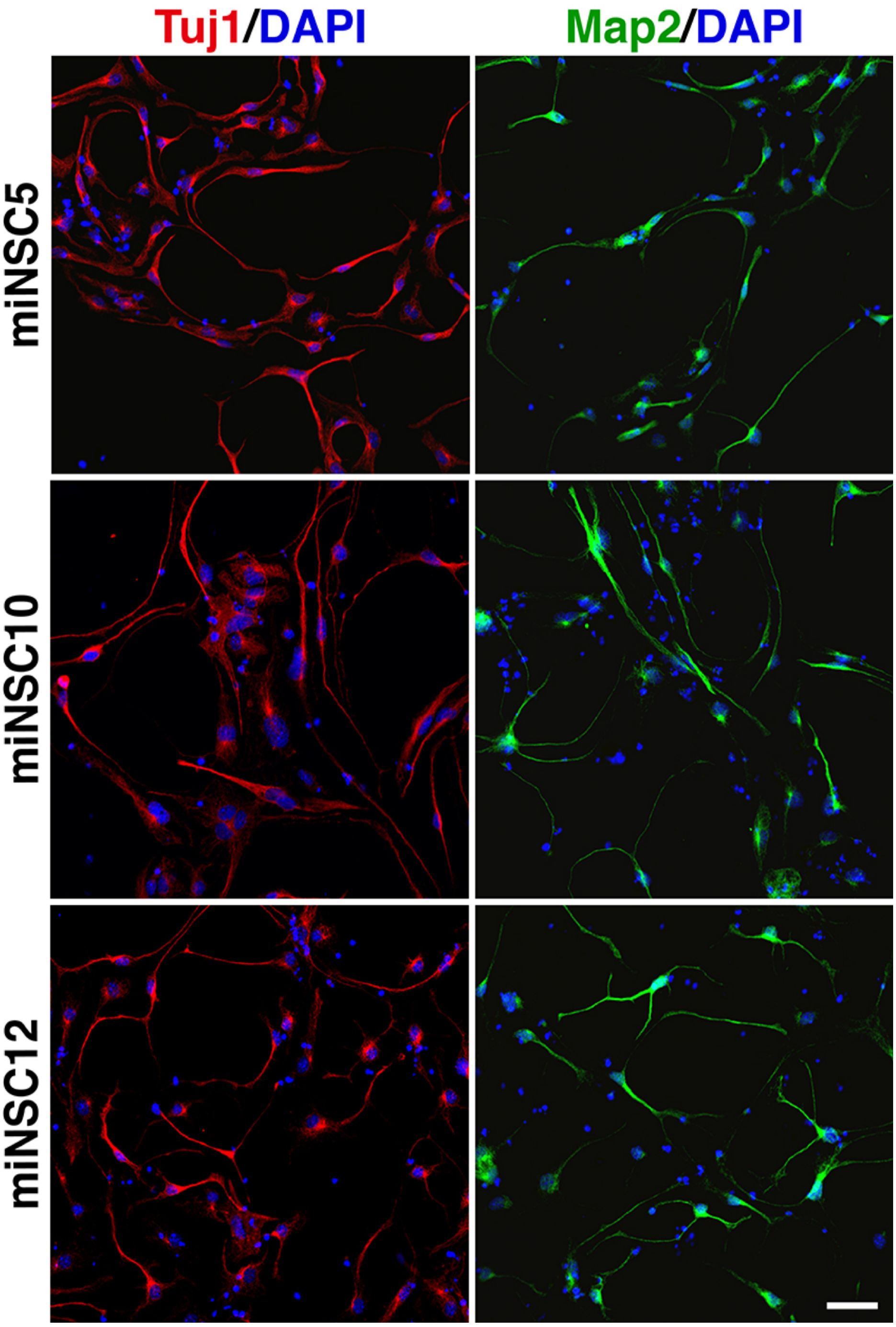

Supplementary Figure 8. Differentiation potential of different iNSC lines derived from MEFs by Ptf1a induction. miNSC5, miNSC10 and miNSC12 cell lines have a similar ability to differentiate into neurons that are immunoreactive for Tuj1 or Map2. Scale bars, 40 μm.

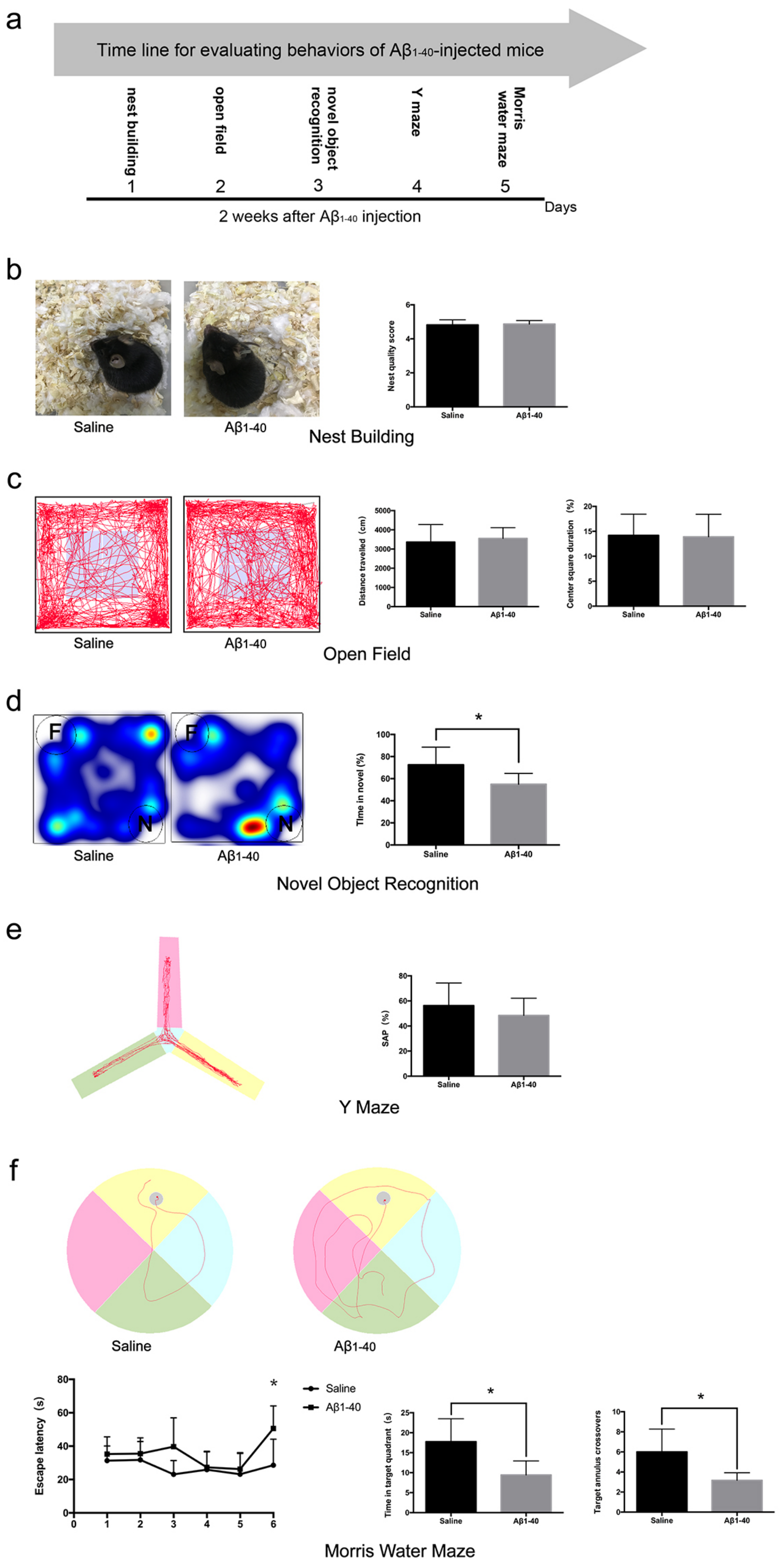
a
Time line for evaluating behaviors of Aβ1-40-injected mice
nest building
open field
novel object recognition
Y maze
Morris water maze
1
2
3
4
5
Days
2 weeks after Aβ1-40 injection
b
Saline
Aβ1-40
Nest quality score
Nest Building
c
Saline
Aβ1-40
Distance travelled (cm)
Center square duration (%)
Open Field
d
F
N
Saline
Aβ1-40
Time in novel (%)
*
Novel Object Recognition
e
SAP (%)
Y Maze
f
Saline
Aβ1-40
Escape latency (s)
Time in target quadrant (s)
Target annulus crossovers
*
Morris Water Maze

Supplementary Figure 9. Injection of A$\beta_{1\text{-}40}$ in the dentate gyrus of the hippocampus caused cognitive impairments in mice. (**a**) Timetable of evaluating the behaviors of A$\beta_{1\text{-}40}$-injected mice. The order for behavioral tests is nest building, open field test, novel object recognition, Y maze test, and Morris water maze test. (**b**) Nest building test. The nest building behavior of saline- and A$\beta_{1\text{-}40}$-injected mice was analyzed by assessing the nest quality after 24-hr exposure to a sheet of tissue cotton. No statistically significant difference was observed between the A$\beta_{1\text{-}40}$-injected group and control group. (**c**) Open field test. An illustrative example of travel pathways for a saline-injected mouse and an A$\beta_{1\text{-}40}$-injected mouse in the open field test. Distance traveled and center square duration in the open field test show no difference between the groups. (**d**) Novel object recognition test. An illustrative example of travel hotspot maps for a saline-injected mouse and an A$\beta_{1\text{-}40}$-injected mouse in the novel object recognition test. A$\beta_{1\text{-}40}$-injected mice show a significant deficit in the preference for the novel. (**e**) Y maze test. An illustrative example of travel pathways for a saline-injected mouse and an A$\beta_{1\text{-}40}$-injected mouse in the Y maze test. Spontaneous alternation (SAP) in the Y maze was unchanged in the A$\beta_{1\text{-}40}$-injected mice. (**f**) Morris Water maze test. An illustrative example of travel pathways for a saline-injected mouse and an A$\beta_{1\text{-}40}$-injected mouse in the Morris Water maze test. Learning curves for Morris water maze acquisition trials were obtained across a period of 6 d. Time spent in target quadrant in the Morris water maze shows that A$\beta_{1\text{-}40}$-injected mice spend less time in the target quadrant. Target annulus crossovers reveal that A$\beta_{1\text{-}40}$-injected mice failed to show a preference for the target platform location. Data are presented as mean ± SD (n=6-11). Asterisks indicate significance in ANOVA test: *$p < 0.05$.

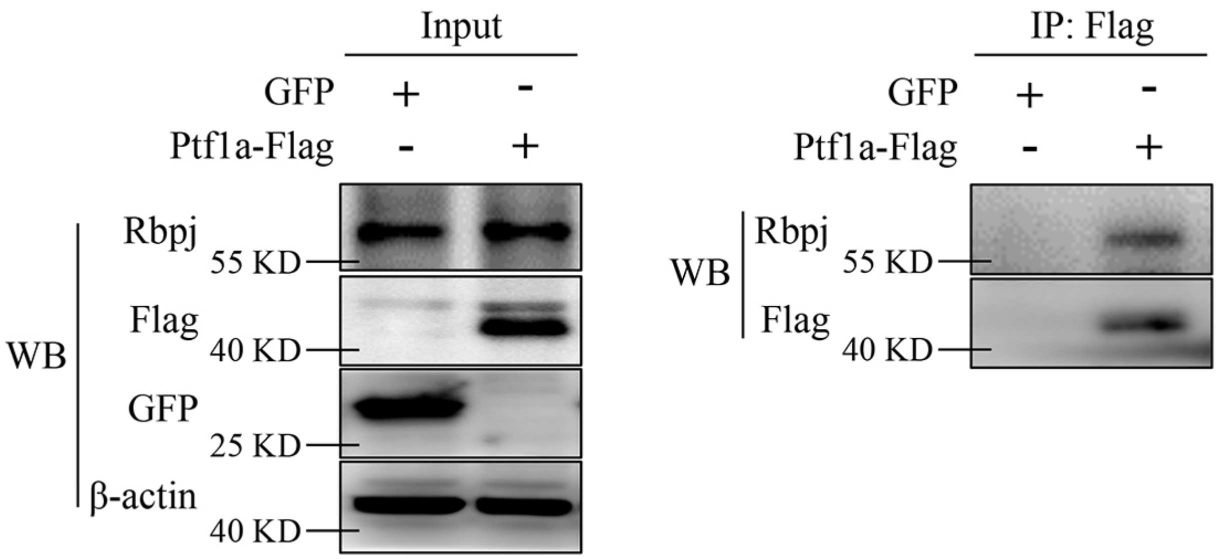

Supplementary Figure 10. Interaction between Ptf1a and Rbpj in MEFs. Cell lysates from MEFs transfected with Flag-tagged Ptf1a or GFP expression plasmids were immunoprecipitated (IP) with an anti-Flag antibody and Western-blotted (WB) with antibodies against Rbpj or Flag. Input proteins were detected by Western blotting with antibodies against Rbpj, Flag or GFP. And equal input of proteins in each sample was confirmed by Western blotting with an anti-β-actin antibody.

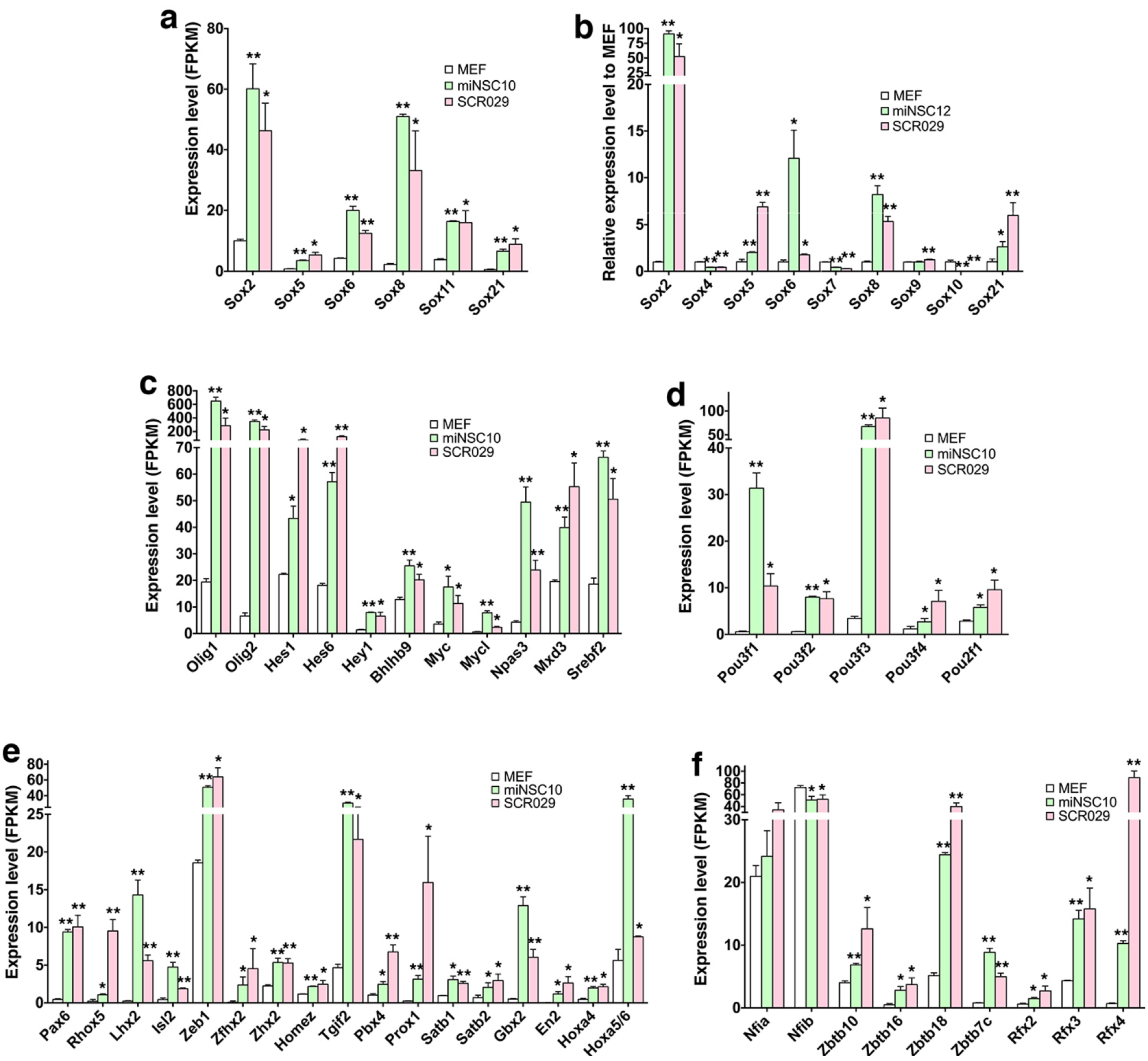

Supplementary Figure 11. Expression levels of several families of TF genes upregulated or highly expressed in both miNSCs and NSCs compared to MEFs. (**a**) Expression levels (FPKM) of significantly upregulated *Sox* genes in miNSC10 and SCR029 cells. (**b**) qRT-PCR analysis of *Sox* family gene expression to validate representative *Sox* genes upregulated in miNSCs and control NSCs. (**c-e**) Expression levels (FPKM) of significantly upregulated bHLH genes (c), POU domain genes (d) and homeobox genes (e) in miNSC10 and SCR029 cells. (**f**) Expression levels (FPKM) of upregulated or highly expressed Nfi genes, zinc finger and BTB domain genes, and Rfx genes in miNSC10 and SCR029 cells. Data are presented as mean ± SD (n=3). Asterisks indicate significance in unpaired two-tailed Student's t-test: *P<0.05, **P<0.001.

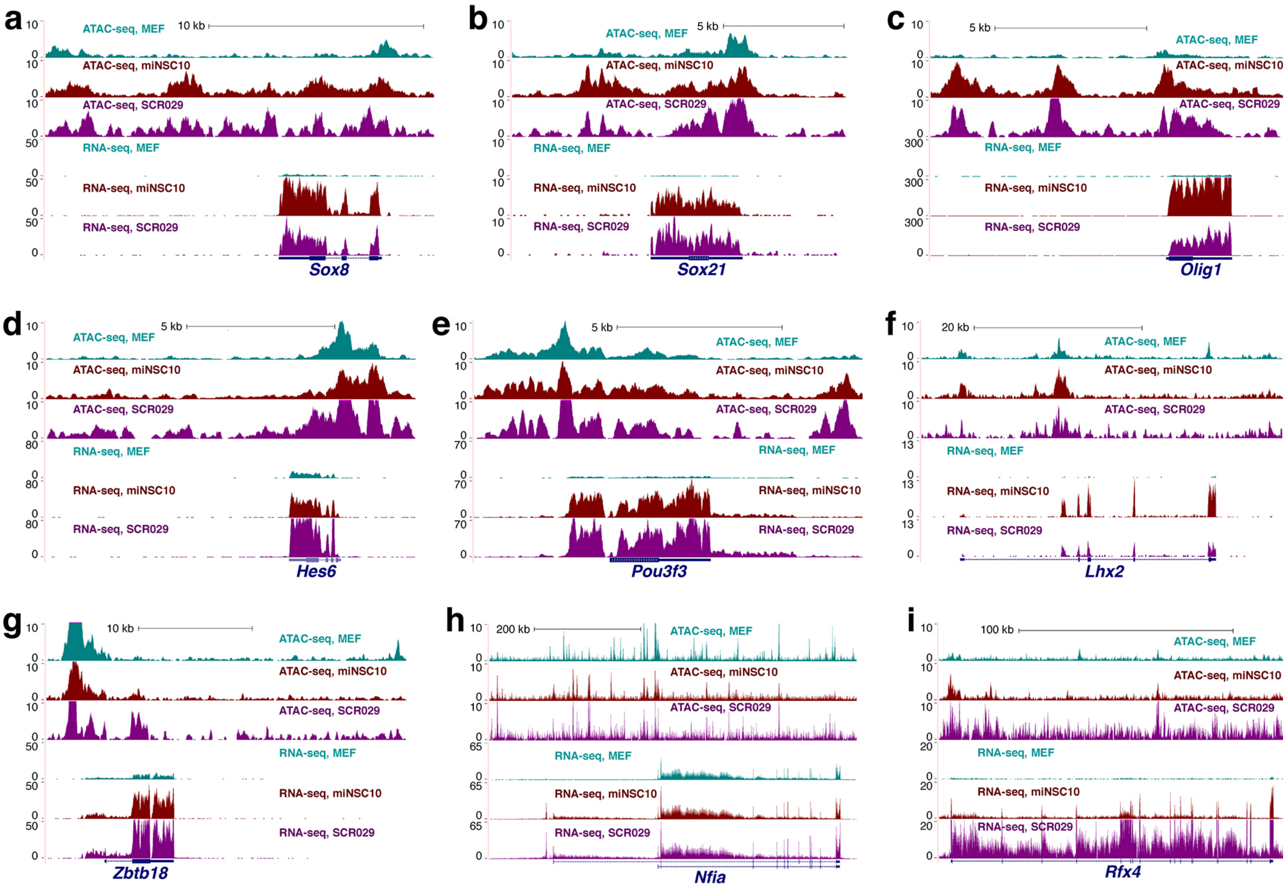

Supplementary Figure 12. ATAC-seq and RNA-seq profiles of representative upregulated TF genes in in both miNSCs and NSCs compared to MEFs. (**a-i**) Genome browser view of ATAC-seq and RNA-seq signals at the *Sox8*, *Sox21*, *Olig1*, *Hes6*, *Pou3f3, Lhx2, Zbtb18, Nfia,* and *Rfx4* loci in MEF, miNSC10 and SCR029 cells. The y axis represents the number of normalized reads.

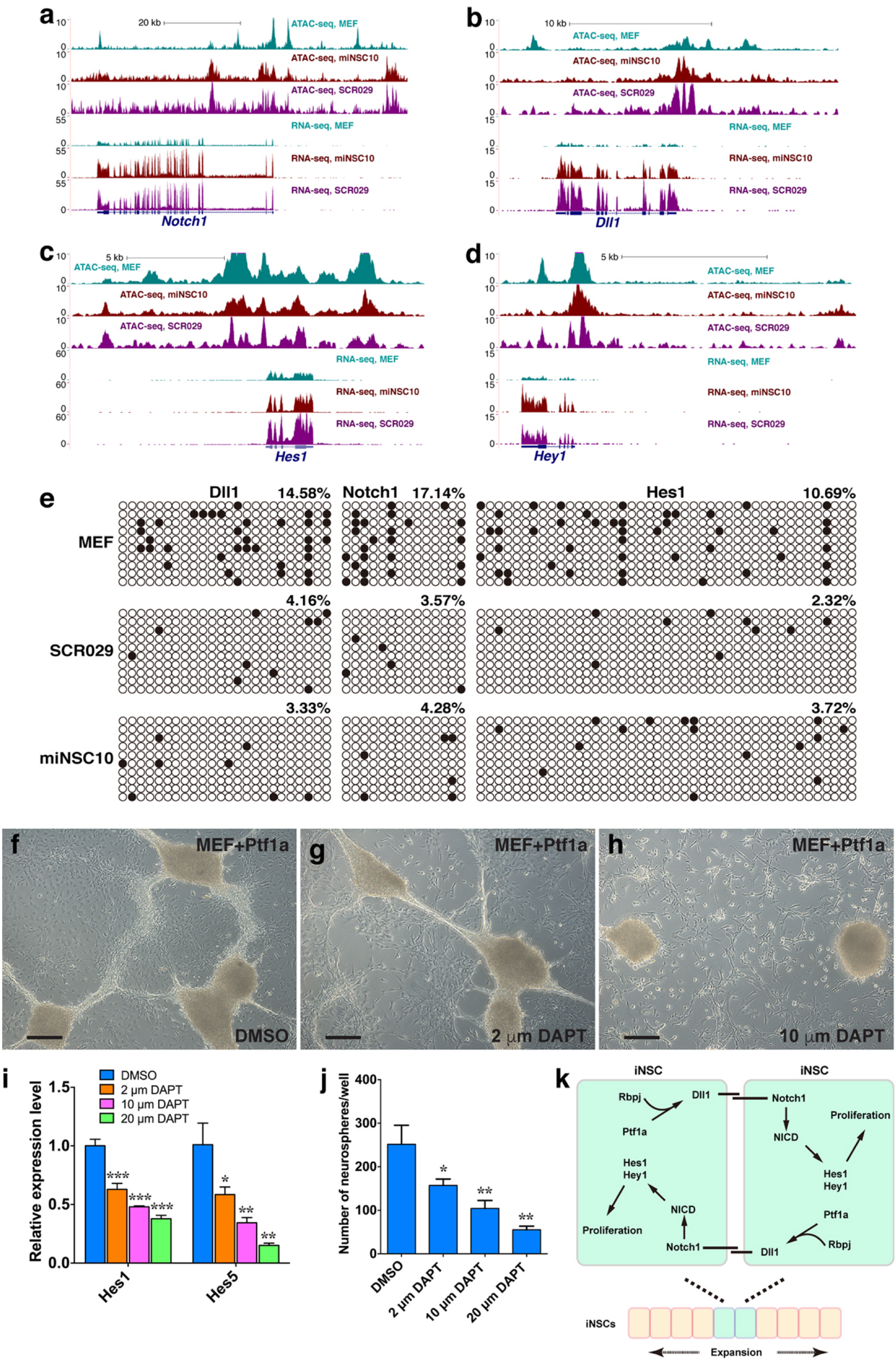
a
ATAC-seq, MEF
ATAC-seq, miNSC10
ATAC-seq, SCR029
RNA-seq, MEF
RNA-seq, miNSC10
RNA-seq, SCR029
Notch1
b
Dll1
c
Hes1
d
Hey1
e
Dll1 14.58%
Notch1 17.14%
Hes1 10.69%
MEF
SCR029
miNSC10
f
g
h
MEF+Ptf1a
DMSO
2 μm DAPT
10 μm DAPT
i
Relative expression level
DMSO
2 μm DAPT
10 μm DAPT
20 μm DAPT
Hes1
Hes5
j
Number of neurospheres/well
k
iNSC
Rbpj
Dll1
Notch1
Ptf1a
NICD
Hes1
Hey1
Proliferation
iNSCs
Expansion

Supplementary Figure 13. Inhibition of Notch signaling causes a decrease in the number of Ptf1a-induced neurospheres. (**a-d**) Genome browser view of ATAC-seq and RNA-seq signals at the *Notch1, Dll1, Hes1* and *Hey1* loci in MEF, miNSC10 and SCR029 cells. The y axis represents the number of normalized reads. (**e**) DNA methylation status was examined in the promoter regions of *Dll1*, *Notch1* and *Hes1* genes. Filled and empty circles represent methylated and unmethylated CpGs, respectively. The percentage of methylation is indicated for each cell type, which is greatly reduced in miNSC10 and SCR029 cells compared to MEFs for all three genes. (**f-h**) Neurosphere formation in MEFs by Ptf1a in the absence or presence of 2 and 10 μm DAPT. (**i**) Relative expression levels of *Hes1* and *Hes5* in the absence or presence of 2, 10 and 20 μm DAPT as determined by qRT-PCR assay. Data are presented as mean ± SD (n=4). Asterisks indicate significance in unpaired two-tailed Student's t-test: *P<0.05, **P<0.005, ***P<0.001. (**j**) Quantification of neurospheres induced by Ptf1a in the absence or presence of 2, 10 and 20 μm DAPT. 4 x 10^4 of MEFs were seeded into each well of 12-well plates, infected with Ptf1a, and cultured with or without DAPT. Neurospheres in each well were counted at day 10 following virus infection. Data are presented as mean ± SD (n=4). Asterisks indicate significance in unpaired two-tailed Student's t-test: *P<0.001, **P<0.0001. (**k**) A feedforward loop to maintain iNSC proliferation and self-renewal. iNSCs may adopt a feedforward loop to promote proliferation and self-renewal in a paracrine manner. The Ptf1a-Rbpj complex binds to the promoter of *Dll1* and promotes its expression. Dll1 binds to the Notch1 receptor on neighbor cells, which causes the release of the Notch intracellular domain (NICD) and leads to activation of *Hes1* and *Hey1* expression. Hes1 and Hey1 in turn activate downstream targets that promote cell proliferation. Scale bars, 160 μm in (**f-h**).

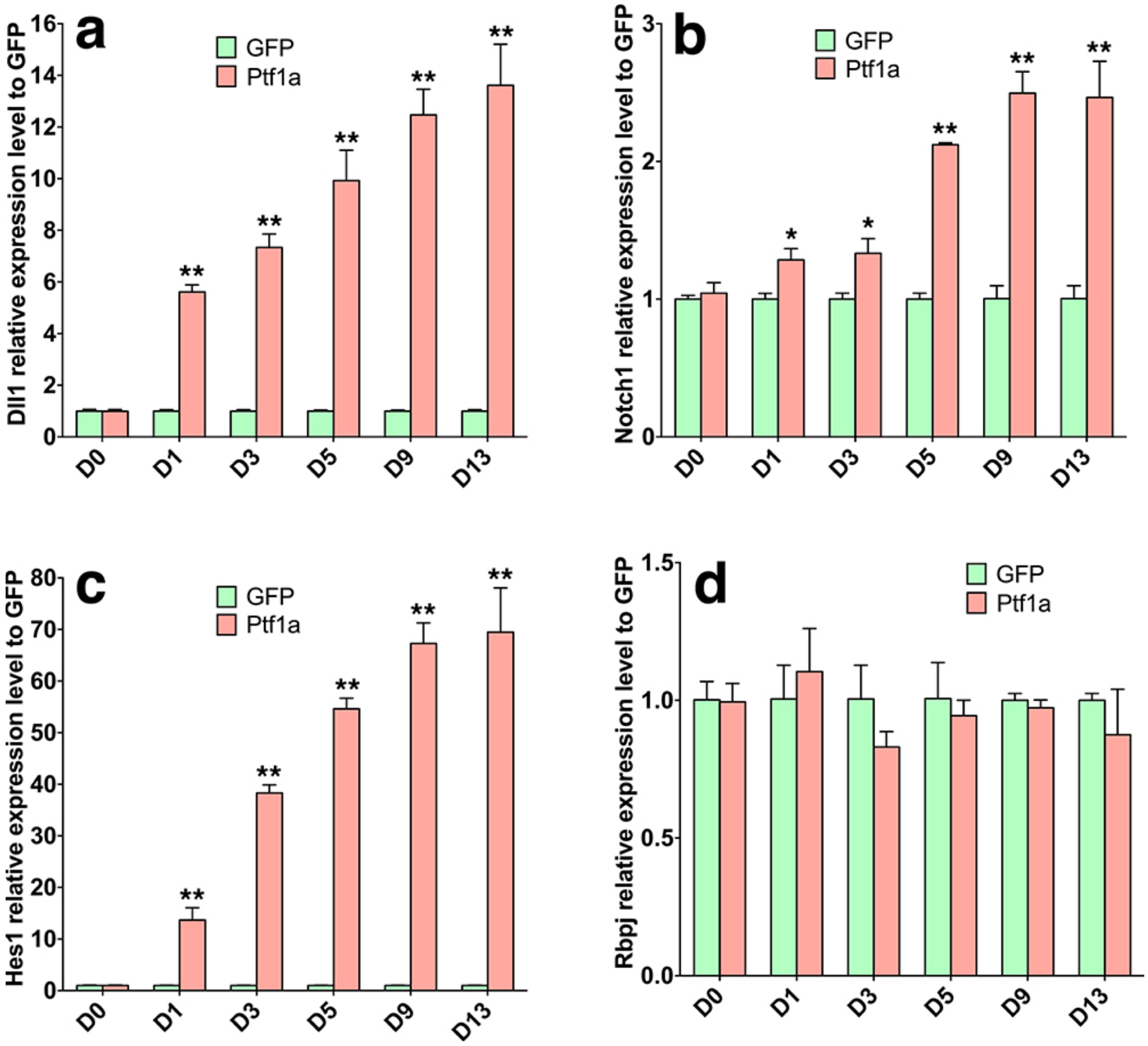

Supplementary Figure 14. Time course of upregulation of Notch-signaling component genes during Ptf1a reprogramming. Compared to MEFs infected with GFP lentiviruses, the expression levels of *Dll1*, *Notch1* and *Hes1* were increased in a time-dependent manner in MEFs infected with Ptf1a viruses (**a-c**), whereas that of *Rbpj* did not change with the culture time (**d**). Data are presented as mean ± SD (n=4). Asterisks indicate significance in unpaired two-tailed Student's t-test: *P<0.005, **P<0.0005.

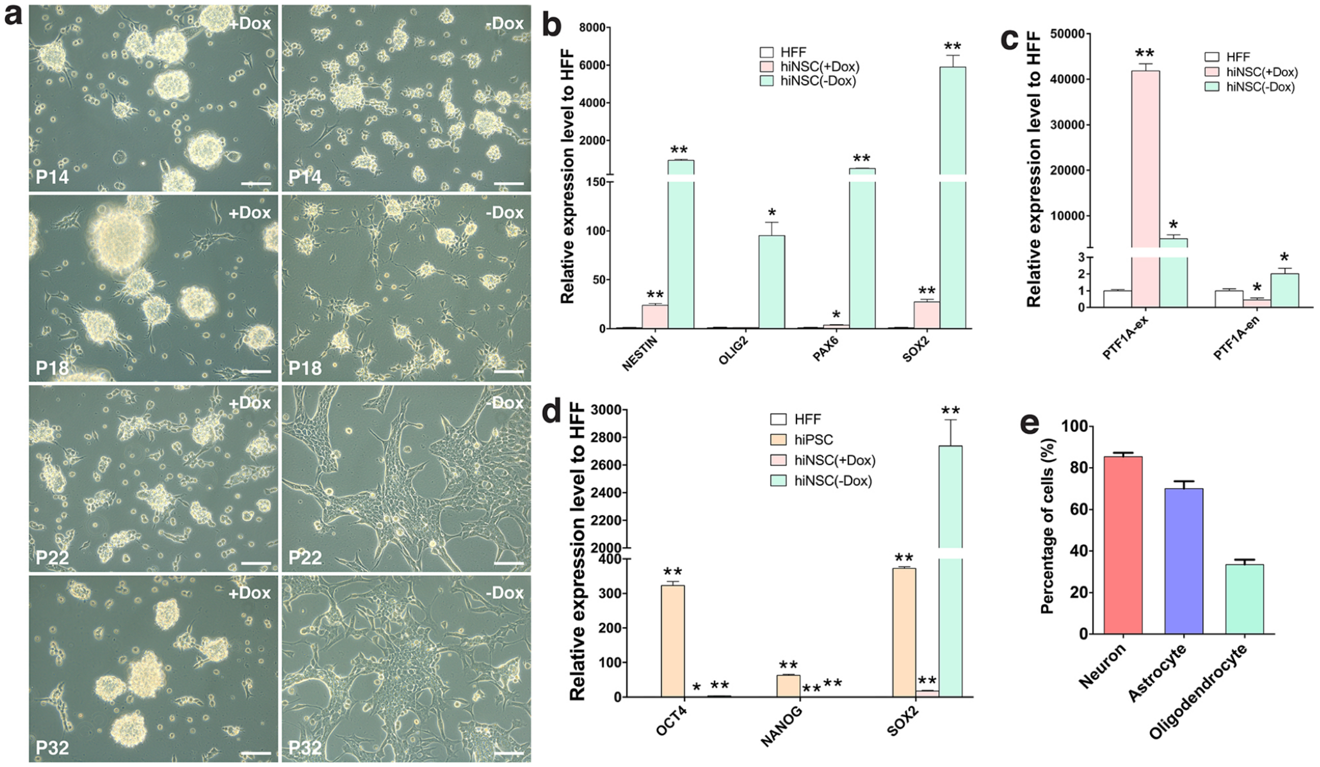

Supplementary Figure 15. Properties of Ptf1a-induced hiNSCs. (**a**) In the presence of doxycycline (Dox), neurosphere cells directly reprogrammed from HFFs by infection of Dox-inducible Ptf1a lentiviruses continued to produce neurospheres up to passage 32 (P32); whereas in the absence of Dox, they ceased to generate neurospheres around passage 20 and became monolayered cells afterwards. (**b**) qRT-PCR assay of the expression levels of *NESTIN*, *OLIG2*, *PAX6* and *SOX2* genes in hiNSCs in the presence or absence of Dox. There is a dramatic increase in expression of these NSC marker genes in the absence of Dox compared to the presence of Dox. Data are presented as mean ± SD (n=3). Asterisks indicate significance in unpaired two-tailed Student′s t-test: *P<0.005, **P<0.0001. (**c**) qRT-PCR analysis of the expression levels of the endogenous (en) and exogenous (ex) *PTF1A* genes in hiNSCs in the presence or absence of Dox. Data are presented as mean ± SD (n=3). Asterisks indicate significance in unpaired two-tailed Student′s t-test: *P<0.01, **P<0.0001. (**d**) qRT-PCR assay showed that compared to hiPSCs, there was no expression of pluripotent factor genes *OCT4* and *NANOG* in hiNSCs. As a pluripotent factor gene and also NSC marker gene, *SOX2* exhibited high levels of expression in hiPSCs and hiNSCs cultured in the absence of Dox. Data are presented as mean ± SD (n=3). Asterisks indicate significance in unpaired two-tailed Student′s t-test: *P<0.05, **P<0.0005. (**e**) Quantification of MAP2+ neurons, GFAP+ astrocytes and O1+ oligodendrocytes differentiated from hiNSCs under different differentiation conditions. Scale bars, 80 μm (**a**).

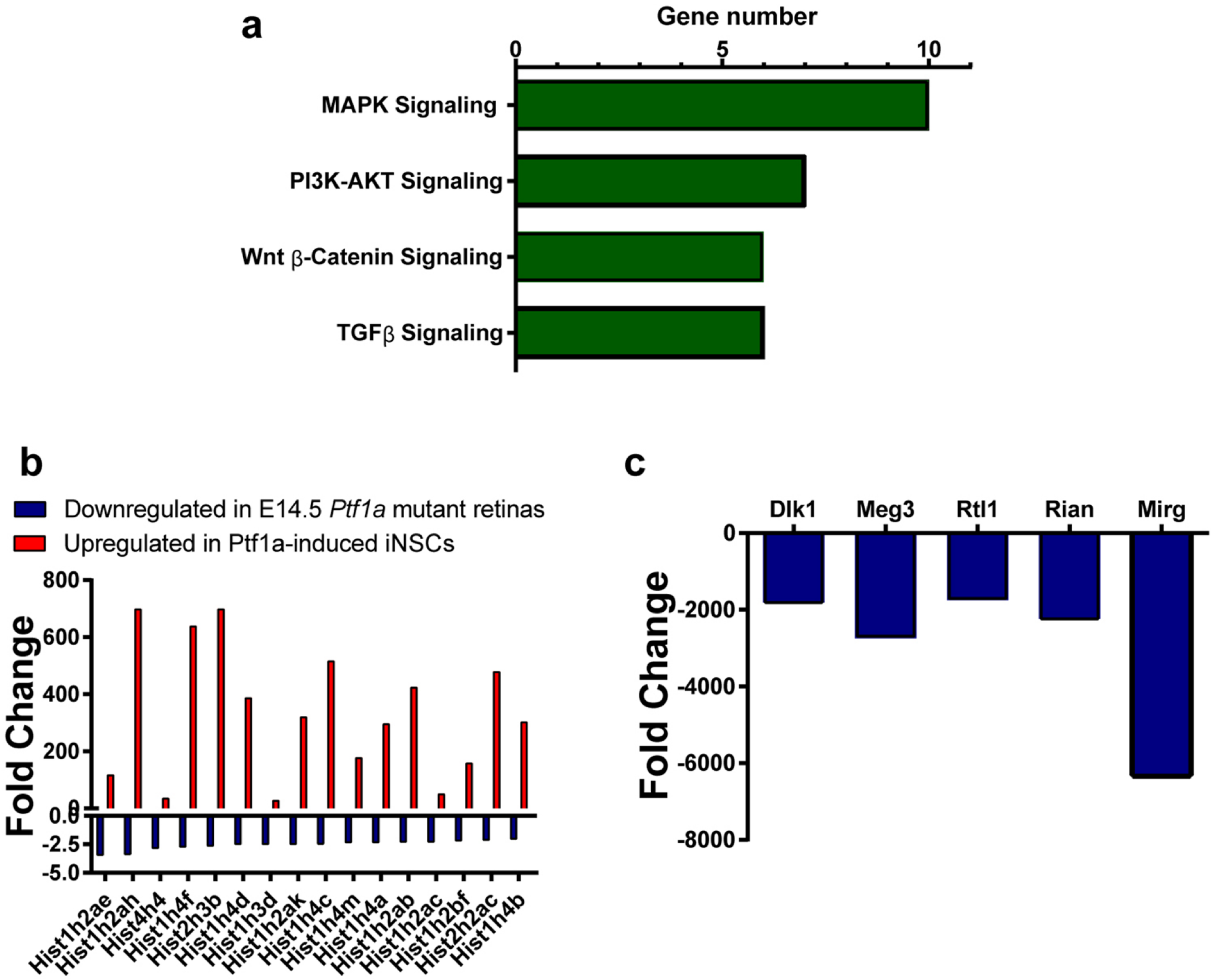

Supplementary Figure 16. Alterations at signaling pathway, epigenetic and posttranscriptional levels during Ptf1a-mediated reprogramming. (**a**) Number of genes upregulated in the PI3K-AKT, Wnt-β-Catenin, MAPK, and TGFβ signaling pathways during reprogramming. (**b**) Histone genes that are downregulated in *Ptf1a*-deficient retinas, but upregulated in Ptf1a-induced iNSCs. (**c**) Dramatic downregulation of members of the Dlk1-Meg3-Rian-Mirg cluster of lncRNAs and microRNAs in iNSCs compared to MEFs. Each column in **b** and **c** represents clustered results from RNA-seq data with 3 biological replicates.
