# Supplementary material for: Direct reprogramming of fibroblasts into neural stem cells by single non-neural progenitor transcription factor Ptf1a
Source: Nat Commun. 2018 Jul 20;9:2865. doi: 10.1038/s41467-018-05209-1 (PMC6054649; doi:10.1038/s41467-018-05209-1)
Supplement: Supplementary file 2 — Description of Additional Supplementary Files [file 41467_2018_5209_MOESM2_ESM.pdf]

Description of Additional Supplementary Files

File Name: Supplementary Data 1
Description: List of genes differentially expressed between miNSC10 and MEF cells as determined by RNA-seq analysis.

File Name: Supplementary Data 2
Description: Gene-specific primers used for RT-PCR analyses.
